# Shade and Drought Shape Stomatal Kinetics in Beech Saplings

**DOI:** 10.1111/ppl.70856

**Published:** 2026-04-03

**Authors:** Yasin Gundesli, Emilie Joetzjer, Oliver Brendel, Didier Le Thiec, David Combemale, Cyril Buré, Matthias Cuntz

**Affiliations:** ^1^ Université de Lorraine, AgroParisTech, INRAE, UMR Silva Nancy France

**Keywords:** drought stress, European beech, light acclimation, potassium deficiency, stomatal dynamics

## Abstract

Stomata and photosynthesis respond at different rates to rapid changes in light availability, resulting in different dynamics of water and carbon fluxes. Here, we investigated how growth conditions, drought, and potassium availability affect the dynamics of stomatal conductance and photosynthesis in response to rapid changes of irradiance in beech saplings ( *Fagus sylvatica* L.). Two greenhouse experiments were conducted, one comparing saplings grown under sunlit conditions to those grown in shaded conditions, and the other testing the effects of edaphic drought and potassium supplementation on saplings grown on potassium‐poor forest soils. When irradiance was changed abruptly, stomatal conductance (g_s_) changed from an initial steady‐state value to a final steady state; this change has two characteristic time constants: a lag time and a response time. Environmental conditions strongly shaped stomatal dynamics. Shade‐grown leaves had reduced changes of steady‐state *g*_s_, but more reactive stomata, characterized by shorter lag and response times. This allowed relatively greater CO_2_ uptake during transient light periods than if they had the response and lag times of sunlit‐grown leaves. Droughted saplings with sufficient potassium also showed reduced *g*_s_ amplitude and shorter lag and response times enhancing conservation of water compared to well‐watered plants. However, potassium‐deficient saplings showed no significant changes in stomatal dynamics regardless of soil water status. Stomata closed about 20% faster than they reopened across all treatments. The differences in stomatal dynamics were not associated with any changes in stomatal size or density, indicating that physiological rather than morphological traits drive the observed plasticity of stomatal responsiveness in European beech.

## 1 Introduction

Stomata enable gas exchange in plants, regulating carbon dioxide intake for photosynthesis and water loss through transpiration by modulating their aperture (Cowan and Farquhar 1977; Farquhar et al. 1980). Both internal factors such as circadian rhythm and external factors, including vapor pressure deficit, soil moisture, temperature, light, and soil mineral composition (particularly potassium content), influence stomatal aperture (Aasamaa and Sõber 2011; Imtiaz et al. 2023). The adjustment of stomatal aperture by guard cells relies on changes in their water content, with turgid cells resulting in fully open stomata (Lawson and Blatt 2014). Osmoregulatory compounds such as potassium, chloride, and malate traverse cell and vacuole membranes via ion channels activated by membrane depolarization induced by proton pumps, enabling water movement (Schroeder et al. 2001). Additionally, water transporters like aquaporins also facilitate water entry into guard cells (Outlaw Jr 2003). Proton pumps, aquaporins, and ion channels require a signalling process for activation (Cochrane and Cochrane 2009). A change in light intensity is one of the signals initiating these responses. Light availability within forest canopies fluctuates throughout the day through gaps and openings due to changing sun angle, cloud movement, and wind‐driven canopy motion (Chazdon 1988). At a sudden decrease in light during the overpassing of a cloud, a so‐called cloudfleck, photosynthesis declines within seconds but stomata close much more slowly and may remain partially open for several minutes, resulting in continued transpiration despite the reduced carbon gain (Vico et al. 2011; McAusland et al. 2016; Vialet‐Chabrand et al. 2017; Deans et al. 2019; Durand et al. 2019). After the cloud has passed, light increases again and photosynthesis activates almost instantly but is constrained by the much slower stomatal dynamics (Way and Pearcy 2012; McAusland et al. 2016). This fundamental difference in response times between photosynthesis and stomatal conductance generates a persistent temporal decoupling. In the framework of recent studies on photosynthetic and stomatal decoupling (McAusland et al. 2016; Vialet‐Chabrand et al. 2017; Lawson and Vialet‐Chabrand 2019; Gerardin et al. 2018; Durand et al. 2019), “stomatal dynamics” specifically describes the slow stomatal response to changes in irradiance, which is used to quantify the temporal mismatch between net assimilation (*A*_net_) and stomatal conductance (*g*_s_). This is different from earlier approaches that inferred the coordination between photosynthesis and stomata from changes in stomatal CO_2_ concentration (C_i_; Küppers and Schneider 1993). The earlier studies investigated induction of photosynthesis from rapid sunflecks (from seconds to rarely minutes, Küppers and Schneider 1993), starting from dark‐adapted leaves, whereas the newer studies focused on fully sun‐induced leaves and the much slower irradiance changes of passing clouds (cloudflecks), reflecting the dynamic stomatal changes from one steady state to another. McAusland et al. (2016) hence showed a transient suboptimal (lower) intrinsic water‐use efficiency (iWUE = *A*_n_/*g*_s_) after changes in light intensity due to the different response times of *A*_n_ and *g*_s_ until both quantities have reached new steady state values.

Stomatal dynamics can vary within and between species. For example, shade‐adapted plants tend to have quicker stomata than sunlit‐adapted species during opening (Deans et al. 2019). At the intraspecific level, while genotype differences play a role (Durand et al. 2019; Eyland et al. 2021), environmental conditions also determine stomatal dynamics (Durand et al. 2022). The variation of stomatal dynamics can be due to the size and density of stomata; larger stomata take longer to reach steady state during opening or closing (Kardiman and Ræbild 2018). According to Raven (2014), larger stomata tend to open more slowly because their guard cells require a greater absolute ion flux, whereas smaller stomata with a larger membrane surface area relative to their volume can support faster ion transport per unit cell volume, and therefore achieve faster opening. Variations in stomatal dynamics may also be attributed to the number of transporters and their sensitivity to membrane depolarization (Nguyen et al. 2023). Transporters sensitive to induced signals can react faster, thus reducing the time needed for stomatal opening or closure (Nguyen et al. 2023). A higher density of ion transporters enables quicker ion and water exchanges, leading to a faster and more efficient stomatal response (Nguyen et al. 2023). Environmental conditions such as light availability, soil moisture stress and nutrient limitation are likely to influence stomatal dynamics. Soil moisture deficit can increase the speed of stomatal responses in some genotypes of poplar (Durand et al. 2019) and tobacco (Gerardin et al. 2018). Rapid stomatal response increases drought tolerance in rice and enhances long‐term water use efficiency (WUE; Qu et al. 2016). In contrast, shade‐grown leaves of the herbaceous species *Nicotiana tabacum* and of woody species ( *Podocarpus macrophyllus* , *Eucalyptus urophylla* and *Capsicum chinense* ) did not show clear differences in stomatal dynamic responses compared with sunlit‐grown leaves (Gerardin et al. 2018; Freitas et al. 2023). Strong potassium deficiency has been shown to reduce maximum stomatal conductance in *Glycine max* (soybean), impair drought resistance in tobacco ( *Nicotiana rustica* ), inhibit stomatal closure, and decrease biomass accumulation in *Eucalyptus* clones (de Souza Mateus et al. 2022; Imtiaz et al. 2023). Olive trees ( *Olea europaea* ) experiencing potassium deficiency exhibited an increase in steady‐state stomatal conductance, as well as dehydration, reduced growth, and reduced WUE (Arquero et al. 2006). While potassium availability is crucial for osmotic adjustment of guard cells, contrasting responses on its impact on stomatal dynamics have been reported in the literature depending on the tree species studied (Imtiaz et al. 2023).

European beech ( *Fagus sylvatica* ) is a predominant deciduous tree species in European forests, covering more than 7.1% of the forested area (Köble and Seufert 2001). Beech trees grow on soils of varying quality and have been observed even on sites with very low potassium levels (Calvaruso et al. 2017). Due to strong droughts in recent years, a substantial decline in beech trees have been observed in some areas of Europe (Martinez del Castillo et al. 2022; Rukh et al. 2023). European beech displays a pronounced gradient in leaf characteristics from the top of the canopy to the shaded forest floor, like a decrease in leaf mass per area (LMA) and corresponding decreases in photosynthetic parameters such as maximum Rubisco carboxylation rate (*V*_cmax_) and maximum electron transport rate (*J*_max_) (Davi et al. 2008; Montpied et al. 2009). Similarly, upper‐canopy leaves display higher stomatal conductance than shaded leaves (Bögelein et al. 2012). These results emphasize that *Fagus sylvatica* shows a strong plasticity in response to light environment. Under drought, beech reduces stomatal conductance, but stomata can reopen rapidly after re‐watering (Kutsch et al. 2004; Lemoine et al. 2002). While steady state stomatal conductance responses to drought and light environment are well documented in beech, much less is known about the dynamics of stomatal adjustment, particularly the speed of closing and opening in response to cloudflecks and how this is modulated by light availability, soil water stress, and nutrient conditions.

We conducted a greenhouse experiment with beech saplings grown under different light and nutrient conditions, and subjected to different water regimes. This setup allowed us to investigate: (i) Do leaves of European beech grown under different light conditions exhibit different stomatal dynamics in shade‐grown leaves, such as slower stomatal responses? (ii) Does soil water deficit induce an increase in the speed of stomatal adjustment in European beech? (iii) How does potassium deficiency alone or in combination with water stress affect stomatal dynamics?

## 2 Materials and Methods

### 2.1 Plant Material and Treatments

**FIGURE 1:**
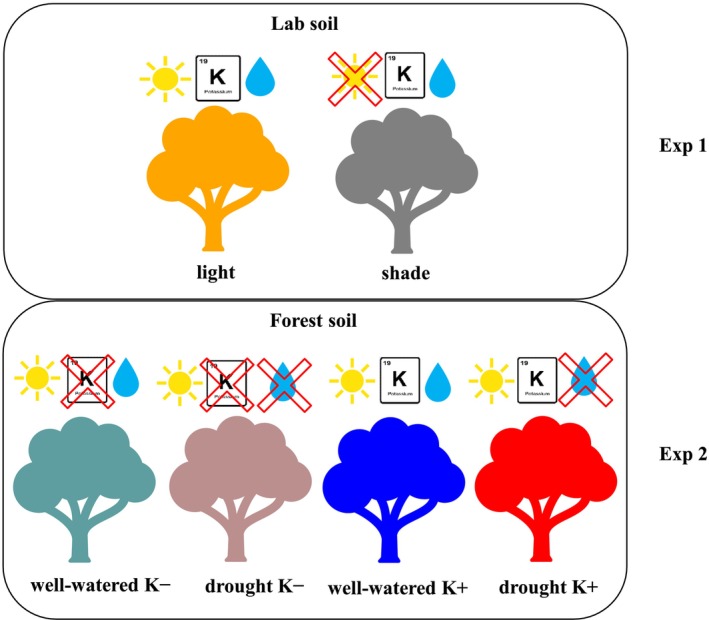
Illustration of the treatments of this study, giving the colour coding used throughout the manuscript. Experiment 1: Orange: Sunlit‐grown plants on fertilised soil (light); grey: Shade‐grown plants on fertilised soil (shade). Experiment 2: Green: Well‐watered plants on forest soil with low K (well‐watered K−); brown: Drought plants on forest soil with low K (drought K−); blue: Well‐watered plants on forest soil with K supplement (well‐watered K+); red: Drought plants on forest soil with K supplement (drought K+).

Two experiments were done on two different groups of 3‐year‐old *Fagus sylvatica* (L.) saplings. In both experiments, saplings were acclimated to greenhouse conditions in 2022 for 1 year, with shade‐grown saplings already growing under shading before the actual experiment in 2023. The greenhouse temperature was maintained below 25°C, with daytime minimums of 15°C and night‐time minimums of 10°C throughout the growing season (see Figure S2 for more details). In winter, the temperature was consistently maintained between 10°C and 15°C every day. The first group of saplings (light/shade, Experiment 1, seen in Figure 1) was obtained from the French national forest service (ONF), from the north‐east provenance of France (FSY201). It consisted of 25 plants that were repotted on 27 March 2022 into 8‐l pots in a mixture of sand, peat, and brown earth in a ratio 2:1:1. On 14 April 2023, the soil was fertilized by adding 14 g of granulated fertilizer (Nutricote T100; 13:13:13 NPK + 2 MgO + micronutrients; Fertil SAS), followed by an additional supplementation of 7 g on 23 June 2023. Within this group, we differentiate two growing treatments referred to as “light” and “shade” (Figure 1, top). Thirteen saplings were exposed to natural light conditions in greenhouse (light), while 12 individuals were placed under shade conditions (shade) during the whole time in the greenhouse. The shade conditions were obtained by building a shadow box covered by an aluminium shade cloth, which shades 75% of the surface (Aluminet 75% shade cloth). The maximum photosynthetic photon flux density (PPFD) in the greenhouse was 1540 μmol m^−2^ s^−1^, while in the shade box, it was 177 μmol m^−2^ s^−1^ measured on 02 June 2023. In the shade box, we obtained 11.34% ± 0.19% (*N* = 6) higher PPFD compared to the PPFD in the surrounding greenhouse. PPFD in the shade condition was measured at six different points in the enclosure and compared to the ambient PPFD of the entire greenhouse. Watering to field capacity was carried out two to three times a week. These conditions were maintained during 2022 and 2023 so that the buds formed at the end of 2022 (for the leaves of 2023) were already formed under shaded/sunlit, well‐watered, and fertilized conditions. The second group consisted of 28 3‐year‐old beech saplings divided into 4 experimental groups (drought × potassium stress, Experiment 2, seen in Figure 1), which originated from a forest at Montiers‐sur‐Saulx in the department Meuse in north‐eastern France (latitude 48°31′54 N, longitude 5°16′08 E). The forest soil has a naturally low potassium (K) content of 0.139 cmolc kg^−1^ (Calvaruso et al. 2017). The saplings were repotted in April 2022 into 8‐l pots in soil stemming from the same forest. White quartz gravel was added on the surface to reduce soil surface evaporation. Plants received a first fertilization immediately after repotting in April 2022, consisting of 43 mg N kg^−1^, 7 mg P kg^−1^, and 7 mg Mg kg^−1^ of substrate. In addition, potassium was supplied at this time to half of the experimental groups at a rate of 985 mg K kg^−1^ of substrate. A second fertilization was applied in June 2022, providing 43 mg N kg^−1^, 22 mg P kg^−1^, and 7 mg Mg kg^−1^ of substrate. No additional potassium was supplied at this stage. As a result, soils of the non‐supplemented groups contained 0.11 cmol₍c₎ kg^−1^ of exchangeable potassium, whereas soils of the potassium‐supplemented groups contained 1.18 cmol₍c₎ kg^−1^ of exchangeable potassium. The drought experiment was run in 2023 using the relative extractable soil water content (REW) as measure of the drought. REW was calculated from soil water content (SWC) as (actual SWC—SWC at wilting point)/(SWC at field capacity—SWC at wilting point), with a mean SWC at field capacity of 29.87% ± 1.43% (*N* = 72) and a SWC at wilting point of 13%. Available soil water content (SWC) was based on a calibration between volumetric SWC measured by Time Domain Reflectometry (Trime Pico‐32, IMKO) and pot weight using the watering robot located at INRAE Champenoux as described by Bogeat‐Triboulot et al. (2019). The experimental targets were 82% REW for control plants and 35% REW for drought plants. This moderate drought level was chosen as it corresponds to a decrease in steady‐state stomatal conductance (Jonard et al. 2011). Half of the plants with potassium supplement were subjected to this experimental soil water deficit. Reduction of soil water content started gradually on day 153 of 2023, reached the control level of 82% REW on day 163 and the drought level of 35% REW from day 173 onwards for 14 days (Figure S3). Green leaf samples from the first flush (first growth) were collected, ground, and analysed for potassium (K) concentrations using a sequential X‐ray fluorescence spectrometer (S8 TIGER 1 kW, Bruker). The upper threshold for nutrient deficiency symptoms for *Fagus sylvatica* is 4.75 g kg^−1^ dry leaf matter, while the lower threshold for normal nutrition is 6.00 g kg^−1^ (Raluca‐Elena et al. 2022). The mean and standard deviation of potassium concentrations measured in the leaves of well‐watered plants from Montiers without K supplement (well‐watered K−) and droughted plants from Montiers without K supplement (drought K−) treatments were 3.61 ± 0.58 g kg^−1^ and 4.92 ± 0.73 g kg^−1^ in our study, respectively, which are close or below the deficiency threshold. Well‐watered plants from Montiers with K supplement (well‐watered K+) and droughted plants from Montiers with K supplement (drought K+) treatments showed 6.81 ± 0.83 g kg^−1^ and 7.70 ± 2.59 g kg^−1^, respectively, indicating sufficient potassium.

We hence had six treatments in total the short names in the parentheses will be used in the following (Figure 1): sunlit‐grown (light) and shade‐grown (shade) plants on a well‐fertilised soil; well‐watered plants on forest soil without K supplementation (i.e., with low K levels; well‐watered K−) and plants on forest soils with K supplement (well‐watered K+); and droughted plants on forest soils without K supplement (drought K−) and with K supplement (drought K+).

### 2.2 Morphological Measurements

Leaves of Experiment 1 and of Experiment 2 were immediately dried after harvesting for determination of leaf mass per area (LMA, see Table 1 for all abbreviations and units) by drying them at 60°C for 48 h. Five fully expanded leaves adjacent to the gas‐exchange leaf at the top of each plant were selected, together with the leaf used for the gas‐exchange measurements. Leaves from the drought‐stressed and potassium‐stressed plants were reserved for other types of analysis (chemical composition) and were not used for LMA measurements. Leaf weight was recorded using a precision balance (METTLER TOLEDO PB303‐S), while leaf area was determined using a flatbed scanner (Epson Perfection V370) and analysed with the open‐source software ImageJ (https://imagej.net/ij/). Leaves used for LMA determination were previously used for relative water content measurements, which required repeated hydration‐dehydration steps, and were therefore not suitable for subsequent chemical analyses.

**TABLE 1:** Symbols and abbreviations used in the text along with their corresponding units.

Parameter	Definition	Unit
*A*_max_	Net CO_2_ assimilation rate at CO_2_ and light saturation	μmol m^−2^ s^−1^
*A*_n_	Net CO_2_ assimilation rate	μmol m^−2^ s^−1^
*A*_n,start_	Initial steady‐state assimilation rate	μmol m^−2^ s^−1^
*A*_n,end_	Final steady‐state assimilation rate	μmol m^−2^ s^−1^
*A*_sat_	Net CO_2_ assimilation rate at 420 ppm of CO_2_ and light saturation	μmol m^−2^ s^−1^
Δ*g*_s_	Amplitude of stomatal conductance	mmol m^−2^ s^−1^
*E*	Transpiration rate	mmol m^−2^ s^−1^
*G*_E_	Water conserved due to delayed stomatal opening	mmol m^−2^
*g*_s_	Stomatal conductance	mol m^−2^ s^−1^
*g*_s,start_	Initial steady‐state stomatal conductance	mol m^−2^ s^−1^
*g*_s,end_	Final steady‐state stomatal conductance	mol m^−2^ s^−1^
*J*_max_	Maximum electron transport rate	μmol m^−2^ s^−1^
*LMA*	Leaf mass per area	g m^−2^
*L*_A_	Limitation of net photosynthesis due to stomatal conductance	μmol m^−2^
*L*_E_	Water lost due to delayed stomatal closure	mmol m^−2^
*L*_E95_	Excess water loss after assimilation reaches 95%	mmol m^−2^
*λ*	Lag time: delay between stimulus and maximum rate of stomatal response	min
*τ*	Response time: time constant	min
*SL*_max_	Maximum slope: maximum rate of change of stomatal conductance	mol m^−2^ s^−2^
*PPFD*	Photosynthetic photon flux density	μmol m^−2^ s^−1^
*R*_d_	Day respiration	μmol m^−2^ s^−1^
*REW*	Relative extractable water	Percentage (%)
SD	Stomatal density	Number per mm^2^
*SL*	Stomatal length	μm
*SW*	Stomatal width	μm
*t*_A95_	Time to reach 95% of final assimilation rate	min
*V*_cmax_	Maximum rate of Rubisco carboxylation	μmol m^−2^ s^−1^
*VPD*_l_	Leaf‐to‐atmosphere vapor pressure deficit	kPa

### 2.3 Measurements of Stomatal Characteristics

Stomatal characteristics, that is, stomatal density (SD), stomatal length (SL), and stomatal width (SW), were measured on the abaxial side of the leaves as beech does not have stomata on the adaxial side (Van Wittenberghe et al. 2012). From each leaf, a circular sample with an area of 78.54 mm^2^ was obtained using a leaf punch. These samples were then imaged using a *Σ*IGMA‐VP scanning electron microscope (Carl Zeiss Microscopy) equipped with a backscattered secondary electron detector. Imaging was conducted at an accelerating voltage of 20 kV, a working distance of 9 mm, and a magnification of ×500, yielding a resolution of 0.279 μm^2^ per pixel. For quantifying stomatal density, we analysed 4 images per leaf sample, each composed of 16 assembled micrographs and covering a combined area of 13.76 mm^2^. We manually counted using ImageJ, excluding any partially visible stomata at the edges of the images. Additionally, stomatal size and length were estimated using ImageJ, with 20 stomata measured per leaf.

### 2.4 Leaf Gas Exchange Measurements

The gas exchange measurements were conducted using a portable LI‐6800 measurement system (LiCor Inc.) with a 3 × 3 cm chamber during the period from mid‐May to mid‐August 2023. The same leaf per individual sapling was used for all gas exchange measurements, representing 42 leaves: 11 light, 9 shade, 7 well‐watered K+, 6 well‐watered K−, 4 drought K+, and 5 drought K−. We ensured balanced sampling by measuring plants across treatments, days, and hours of the day in a randomized order. To test whether stomatal dynamic parameters were affected by the time of day or measurement date, we performed ANCOVAs for each parameter (Δ*g*_s_, *λ*, *τ*, *SL*_max_), including hour of day (binned) and date as categorical factors. No significant effects of hour of day, date, or their interaction were detected for any parameter, indicating that stomatal dynamics were not influenced by measurement timing under our experimental conditions. We measured between four and six leaves per day, depending on environmental stability and instrument availability. A‐C_i_ curves were generated using the rapid A‐C_i_ response technique (A‐C_i_ dynamics) describe by Stinziano et al. (2017) to estimate the parameters *V*_cmax_ and *J*_max_ of the model by Farquhar et al. (1980). *V*_cmax_ is the maximum rate of Rubisco activity and *J*_max_ is the maximum electron transport rate. The dynamic A‐C_i_ technique is rapid (approximately 10 min per curve), which helps minimize stomatal closure during high CO₂ exposure. They were performed in the morning between 8:00 h and 12:00 h using two LI‐6800 instruments. Leaves were first acclimated to 400 ppm of CO_2_ until stabilisation. Then CO_2_ concentration was increased to 2000 ppm, followed by a gradual decrease to 50 ppm, measured every 5 s under conditions of saturating photosynthetically active radiation (PPFD, 1200 μmol m^−2^ s^−1^) with leaf temperature set to 25°C, leaf‐to‐atmosphere vapor pressure deficit (VPD_l_) set to 1.2 kPa, and a flow rate of 500 μmol s^−1^. We selected a ramp rate of 200 ppm s^−1^ CO_2_, based on preliminary tests, to avoid artifacts such as oscillations in the A‐Ci curves caused by excessively high ramp rates. To ensure that 1200 μmol m^−2^ s^−1^ of PPFD was truly a saturating light intensity, we conducted an additional experiment in which PPFD was gradually decreased from 2000 μmol m^−2^ s^−1^ in steps of 100 μmol m^−2^ s^−1^ down to 50 μmol m^−2^ s^−1^, to observe the point at which assimilation started to decrease for four plants from light‐ and shade‐adapted groups. We employed the slope–intercept regression following the Laisk method to measure CO_2_ compensation point Γ* and day respiration *R*_d_ (Walker and Ort 2015). Three A‐Ci curves were measured with low CO_2_ concentrations ranging from 134 ppm to 5 ppm under three different low light conditions (137, 81, and 49 μmol m^−2^ s^−1^). The value of *R*_d_ was determined as the measured negative net CO_2_ assimilation at the point where the three curves intersect on the y‐axis, while the value of Γ* is the corresponding leaf internal CO_2_ concentration. We only used three curves because the fourth curve, at 225 μmol m^−2^ s^−1^ of PPFD, did not intersect with the other three.

The protocol for testing stomatal dynamics represents a simulation of a cloudfleck: a leaf acclimated to high light will be step‐changed to low light (a passing cloud) and back to high light, thereby inducing first a stomatal closure and then a stomatal (re‐)opening. Preliminary tests were needed to define the range and intensity of the irradiance step change to estimate stomatal conductance (*g*_s_) dynamics as shade‐grown leaves showed photo‐inhibition effects when PPFD values exceed 800 μmol m^−2^ s^−1^. For one leaf of each treatment, we started at 1200 μmol m^−2^ s^−1^ of PPFD and gradually reduced the radiation intensity by steps of 100 μmol m^−2^ s^−1^, waiting for the parameters and stomatal conductance to become stable. Sunlit‐grown plants started to respond at 700 μmol m^−2^ s^−1^ of PPFD and the shade‐grown leaves at 400 μmol m^−2^ s^−1^. We therefore used steps from 400 to 100 μmol m^−2^ s^−1^ of PPFD for stomatal closure and from 100 to 400 μmol m^−2^ s^−1^ of PPFD for stomatal opening. *g*_s_ dynamic responses to changes in irradiance were measured using two‐step changes: (i) Once the leaf was acclimated to the conditions of the Licor chamber with stable values of *A*_n_ and *g*_s_ at 400 μmol m^−2^ s^−1^ of PPFD, stomatal closure was induced by decreasing irradiance to 100 μmol m^−2^ s^−1^ of PPFD. (ii) Once stomatal conductance was stable again at 100 μmol m^−2^ s^−1^, stomatal opening was induced by increasing irradiance to 400 μmol m^−2^ s^−1^ of PPFD. Experiments were performed only during the day from 8:00 h to 18:00 h. VPD_l_ was set to 1.2 kPa, leaf temperature to 25°C, flow rate to 500 μmol s^−1^, and CO_2_ concentration to 420 ppm. The blue‐red light ratio was fixed at 90% red light and 10% blue light. It took, on average, 1.5 h to complete one measurement cycle, including closure and opening phases. To avoid low *g*_s_ at the start of the experiments on cloudy days when ambient PPFD in the greenhouse was low (less than 400 μmol m^−2^ s^−1^), we used an LED panel (Multi‐Color LED FYTO‐Panel–version C, PSI [Photon Systems Instruments]) on the top of the greenhouse with red LEDs of 618–630 nm, blue LEDs of 450–460 nm, and white LEDs at 6500 K, leading to light intensities from 300 to 600 μmol m^−2^ s^−1^ on the leaf surfaces depending on the size of the plant and its distance from the LED panel.

### 2.5 Non‐Steady State Stomatal and Photosynthesis Response

We use a sigmoid Gompertz‐type function to describe the non‐steady‐state stomatal conductance *g*_s_ in response to a step change in light intensity. This model, originally developed by Vialet‐Chabrand et al. (2013) and later modified by Gerardin et al. (2018), was fitted to *g*_s_ measurements following irradiance step‐changes:

(1) $$g_{\text{s}}=g_{\text{s,start}}+\left(g_{\text{s,end}}-g_{\text{s,start}}\right)e^{-e^{\frac{\lambda -t}{\tau }}}$$

where *g*_s_ is the non‐steady‐state stomatal conductance (mol m^−2^ s^−1^), *g*_s,start_ is the initial steady‐state stomatal conductance, *g*_s,end_ is the final steady‐state stomatal conductance (mol m^−2^ s^−1^), *t* is time (min), lag time (*λ*) (min), which is the delay between light changes and the peak rate of change of stomatal conductance, and the response time (*τ*) (min), which is a time constant related to the maximum slope.

Additional dynamic variables were calculated from the estimated dynamic parameters: the maximum slope (*SL*_max_) (mmol m^−2^ s^−2^), which corresponds to the rate of change in stomatal conductance at the lag time *λ* (Equation 3); the amplitude (Δ*g*_s_) (mol m^−2^ s^−1^), defined as the change in stomatal conductance between the final value (*g*_s,end_) and the initial steady‐state value (*g*_s,start_). We further calculated *t*_95_ (min), the time required for *g*_s_ to reach 95% of *g*_s,end_ following a step change in irradiance (Equation 4). Note that (Equation 1) is not a simple logistic function but a Gompertz function. The double‐exponential makes the curve asymmetric around its point of steepest ascent or descent.

The sigmoid model can also be used to fit non‐steady state assimilation (*A*_n_).

(2) $$A_{\text{n}}=A_{\text{n},\text{start}}+\left(A_{\text{n},\mathrm{end}}-A_{\text{n},\text{start}}\right)e^{-e^{\frac{\lambda_{A}‐t}{\tau_{A}}}}$$

The slope of stomatal conductance at time *t* = *λ* (*SL*_max_) is given by:

(3) $${\mathrm{SL}}_{\max}=\frac{\Delta g_{\text{s}}}{\tau \cdot e}$$

with Δ*g*_s_ = *g*_s,end_ − *g*_s,start_ and the Euler number e. *A*_n_ is the non‐steady‐state CO_2_ assimilation rate (μmol m^−2^ s^−1^), *A*_n,start_ is the initial steady‐state assimilation rate and *A*_n,end_ is the final steady‐state assimilation rate (μmol m^−2^ s^−1^), *t* is time (min), *λ*_A_ is the lag time (min), and *τ*_A_ is the response time (min).

**FIGURE 2:**
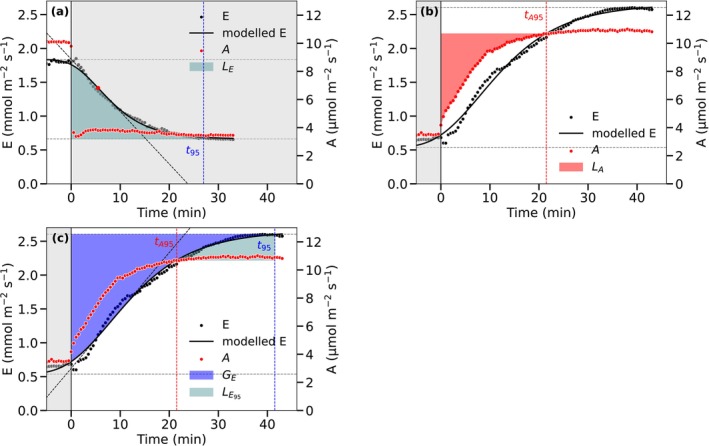
Stomatal response to a step change in irradiance from 400 to 100 μmol m^−2^ s^−1^ (a) and from 100 to 400 μmol m^−2^ s^−1^ (b, c). (a) Water lost during delayed stomatal closure after the decrease in light (cyan area, *L*_E_). (b) Water conserved due to the delayed stomatal opening (purple area, *G*_E_) and excess water loss due to stomatal overshoot after assimilation reaches 95% (cyan area, *L*_E95_). (c) Net assimilation limited due to delayed stomatal opening (red area, *L*_A_).

The time needed to reach 95% of the new steady‐state value for stomatal conductance (*t*_95_) and photosynthetic assimilation (*t*_A95_) are (Figure 2):

(4) $$t_{95}=\lambda -\ln\left(-\ln\left(0.95\right)\right)\cdot \tau \approx \lambda +2.97\cdot \tau$$

(5) $$t_{\text{A}95}=\lambda_{\text{A}}-\ln\left(-\ln\left(0.95\right)\right)\cdot \tau_{\text{A}}\approx \lambda_{\text{A}}+2.97\cdot \tau$$

We employed the function *curvefit* from SciPy v1.12.0 (www.scipy.org) in Python to fit the parameters of the Gompertz function.

The excess water loss (*L*_E_) during delayed stomatal closure was calculated as the cumulative difference between measured transpiration (*E*) and steady‐state transpiration ($E_{\mathrm{end}}^{c}$) after stomatal closure (Figure 2a):

(6) $$L_{E}=\int_{t_{0}}^{t_{95}}\left(E-E_{\mathrm{end}}^{c}\right)\mathrm{dt}$$

The stomatal limitation (*L*_A_) of net photosynthesis (*A*_n_) following a step increase in irradiance was calculated as the cumulative difference between the measured *A*_n_ and final steady‐state assimilation rate *A* ^o^_n,end_, that is, assuming instantaneous stomatal opening. The calculation integrates from the time of the step change (*t*_0_) until stomatal conductance reaches 95% of its final steady‐state value (*t*_95_) (McAusland et al. 2016). This approach quantifies the “lost” photosynthesis due to delayed stomatal opening (Figure 2c):

(7) $$\;L_{\text{A}}=\int_{t_{0}}^{t_{95}}\left(A_{\text{n},\mathrm{end}}^{o}-A_{\text{n}}\right)\mathrm{dt}$$

We define the difference between the final steady‐state transpiration $E_{\mathrm{end}}^{o}$ and actual transpiration during stomatal opening as a theoretical water gain (*G*_E_) (Figure 2b):

(8) $$G_{E}=\int_{t_{0}}^{t_{95}}\left(E_{\mathrm{end}}^{o}-E\right)\mathrm{dt}$$

Stomata continue opening even after assimilation reached a new steady state. This excess water loss due to stomatal overshoot (*L*_E95_) was calculated as actual transpiration up to reaching 95% of its new steady‐state value (*t*_95_, Equation 4) minus the transpiration at the time assimilation reached 95% of its new steady‐state value (*t*_A95_, Equation 5) (Figure 2b):

(9) $$L_{\text{E}95}=\int_{t_{0}}^{t_{95}}\left(E-E\left(t_{\text{A}95}\right)\right)\mathrm{dt}$$

### 2.6 Statistical Analyses

Python packages SciPy v1.12.0 and statsmodels 0.14.1 (www.statsmodels.org) were used for statistical analyses. To test for significant differences between shade‐ and sunlit‐grown leaves (light: *n* = 11, shade: *n* = 9), averages were compared using a two‐sided *t*‐test from SciPy. A one‐sided *t*‐test against a constant (ratio of 1) was used from SciPy to compare the closure/opening ratios of different parameters. A two‐way analysis of variance (ANOVA) was conducted using statsmodels to compare the effects of water stress (well‐watered vs. drought) and potassium level (K+ vs. K−) and their interactions (well‐watered K+: *n* = 7, well‐watered K−: *n* = 6, drought K+: *n* = 4, and drought K−: *n* = 5). *Post hoc* comparisons were performed using Tukey's Honestly Significant Difference (HSD) test from statsmodels. Type 2 linear regressions, accounting for uncertainties in both x‐ and y‐values, were carried out using orthogonal distance regression in SciPy. It is important to acknowledge that the relatively small number of replicates within each treatment group for the water and potassium experiments could limit the statistical power of the ANOVA and post hoc tests. A logarithmic transformation was applied to the percentage values to improve their suitability for statistical testing.

## 3 Results

### 3.1 Variability of Stomatal Responses to Light Changes Between Individual Leaves

**FIGURE 3:**
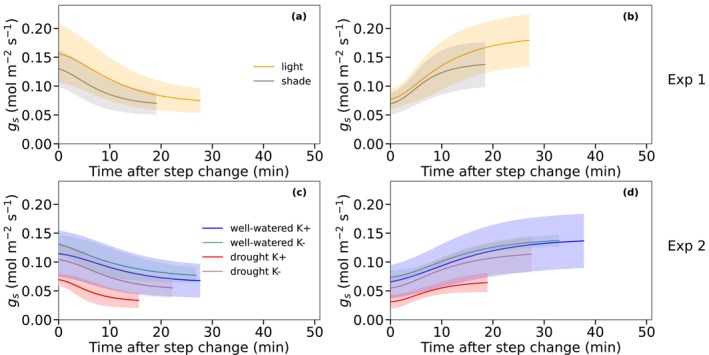
Fitted response curves of stomatal conductance *g*_s_ to step changes of irradiance from 400 μmol m^−2^ s^−1^ to 100 μmol m^−2^ s^−1^ PPFD (a, c) and from 100 to 400 μmol m^−2^ s^−1^ (b, d). The upper row (a, b) shows mean *g*_s_ responses per treatment for Experiment 1 (light vs. shade) and the lower row (c, d) for Experiment 2 (drought and potassium deficiency). Shaded areas represent standard deviations. The beginning of the lines is at the step change of PPFD while the end is at 95% of the total amplitude change (*t*_95_). The number of leaves per treatment was *n* = 11 for light, *n* = 9 for shade, *n* = 7 for well‐watered K+, *n* = 6 for well‐watered K−, *n* = 4 for drought K+, and *n* = 5 for drought K−.

In Experiment 1, both sunlit‐ and shade‐grown leaves showed a gradual decrease in stomatal conductance (*g*_s_) following a decrease in irradiance from 400 to 100 μmol m^−2^ s^−1^ of PPFD (Figure 3a) and a progressive increase after light restoration from 100 to 400 μmol m^−2^ s^−1^ of PPFD (Figure 3b). Sunlit‐grown leaves displayed higher maximum steady‐state *g*_s_ and larger Δ*g*_s_ compared to shade‐grown leaves despite similar minimum *g*_s_ after stomata closure (Figure 3a,b). However, shade‐grown leaves reached 95% of their total conductance change (*t*_95_) in a shorter time, indicating faster stomatal adjustment despite their lower overall conductance (Figure 3a,b). The standard deviation was relatively large for both treatments, revealing substantial variability among individual leaves within each light condition (Figure 3a,b). Stomata of all treatments in Experiment 2 responded to both light decrease and subsequent restoration (Figure 3c,d). In drought K+ plants, both maximum *g*_s_ and minimal *g*_s_ after closure were reduced relative to well‐watered K+ treatments (Figure 3c,d). Well‐watered K+ plants exhibited longer *g*_s_ responses while drought K+ plants reached steady‐state *g*_s_ more rapidly than well‐watered K+ (Figure 3c,d), suggesting faster response of stomata under drought. In contrast, potassium deficiency K– had very little effect on stomatal dynamics in well‐watered plants. Drought K– plants also showed similar responses to the well‐watered plants in contrast to drought K+ plants (Figure 3c,d). Standard deviations were again high across treatments, reflecting a strong heterogeneity in stomatal behaviour among individual leaves. Anatomical factors do not appear to have influenced the observed variation in stomatal conductance. No significant differences were found in stomatal density (SD), stomatal length (SL), or stomatal width (SW) across treatments, whether for shade, drought, or K supplementation (Table S7). Leaf mass per area (LMA) was significantly higher in light‐grown leaves 61.32 ± 2.18 g m^−2^ compared to shade‐grown leaves 46.47 ± 2.30 g m^−2^ (Table S7). In contrast, LMA did not differ across treatments in Experiment 2 (Table S7). Structural leaf traits such as LMA are primarily determined during leaf development and are generally unresponsive to sudden short‐term stress once leaves are fully expanded (Poorter et al. 2009).

### 3.2 Amplitude and Time to Reach 95% of the Change in Stomatal Conductance

**FIGURE 4:**
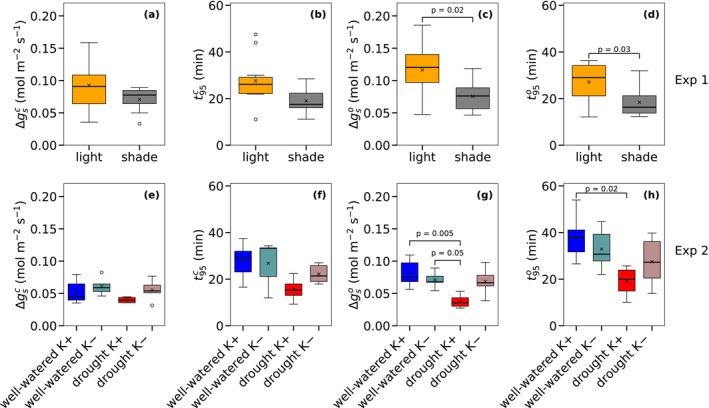
Comparison of the amplitude between initial and final steady‐state *g*_s_ after step changes of incident light (Δ*g*_s_) and the time to reach 95% of Δ*g*_s_ (*t*_95_) during stomatal closure (superscript *c*: a, b) and opening (superscript *o*: c, d) between sunlit‐ and shade‐grown leaves (Experiment 1) and during closure (superscript *c*: e, f) and opening (superscript *o*: g, h) among the different treatments of Experiment 2. Sample sizes and colours are as in (Figure 3). The boxplots are delineated by quartiles. Horizontal lines within the boxes represent the median and crosses represent the mean values. A *t*‐test was conducted between shade‐ and sunlit‐grown leaves (Experiment 1). A two‐way ANOVA with post hoc Tukey tests was performed to compare the means among the groups of well‐watered K+, well‐watered K−, drought K+, and drought K− (Experiment 2). Only *p* < 0.05 are shown in the panels.

Both the amplitude of the steady‐state stomatal conductance changes (Δ*g*_s_) and the time required to reach 95% of this amplitude (*t*_95_) showed only a non‐significant decrease in shade‐grown leaves compared to light‐grown leaves during stomatal closure (Figure 4a,b; Table S3). They were, however, significantly lower in shade‐grown leaves during stomatal opening (Figure 4c,d; Table S2). In Experiment 2, well‐watered K+ and drought K+ plants also showed non‐significant lower Δ*g*_s_ and *t*_95_ in droughted plants during stomatal closing, but significantly smaller Δ*g*_s_ and *t*_95_ in droughted plants during stomatal opening (Figure 4e–h). Potassium deficiency (K−) partly impaired drought‐induced responses: drought K− had non‐significant larger Δ*g*_s_ and *t*_95_ than drought K+ during stomatal closing and opening (Figure 4e–h). The differences in Δ*g*_s_ and *t*₉₅ between well‐watered and droughted plants were hence only significant in the presence of adequate potassium supply (K+). Although stomatal conductance was higher in light‐grown leaves than in shade‐grown leaves in Experiment 1, and lower in the drought K+ treatment compared to the other treatments in Experiment 2, photosynthetic capacities (*A*_max_, *A*_sat_, *V*_cmax_, and *J*_max_) did not show corresponding differences. None of the parameters were higher in light‐grown leaves compared to shade‐grown leaves, nor lower in the drought K+ treatment compared to well‐watered K+ treatment (Table S6). Likewise, Γ* and *R*_d_ remained constant across treatments in both Experiment 1 and 2 (Table S6).

### 3.3 Parameters of the Non‐Steady State Response of Stomatal Conductance

**FIGURE 5:**
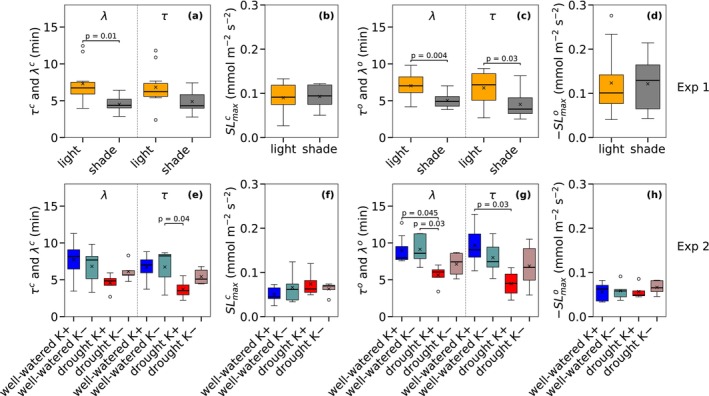
Comparison of lag time (*λ*), response time (*τ*), and the maximum slope (*SL*_max_) during stomatal closure (superscript *c*: a, b) and opening (superscript *o*: c, d) between sunlit‐ and shade‐grown leaves (Experiment 1) and during closure (superscript *c*: e, f) and opening (superscript *o*: g, h) among the different treatments of Experiment 2. The parameters *λ* and *τ* were obtained by fitting the Gompertz model (Equation 1) to the observations. Sample size and colours are as in Figures 3 and 4, and the boxplots follow the same convention as in Figure 4. The same statistical tests as in (Figure 4) were applied.

Contrary to Δ*g*_s_ and *t*_95_, shade‐grown leaves exhibited significantly shorter lag time (*λ*) also during stomatal closure (Figure 5a), while a large variability in the response time (*τ*) prevented it from also being significantly shorter. Both *λ* and *τ* of shade‐grown leaves were, however, significantly shorter compared to sunlit‐grown leaves during stomatal opening (Figure 5c; Table S2). The maximum slope *SL*_max_, on the other hand, was very similar across light and shade treatments as well as during closing and opening (Figure 5b,d). *SL*_max_ is proportional to the ratio of Δ*g*_s_ to *τ* (Equation 3), so that the decrease of the latter for shade‐grown leaves cancelled out the decrease of the former. For Experiment 2, a similar pattern to that observed for Δ*g*_s_ and *t*₉₅ was found for *λ* and *τ* in the water and potassium deficiency treatments (Experiment 2). Both *λ* and *τ* were significantly longer in well‐watered K+ plants than in drought K+ plants during stomatal opening. An exception was observed in the drought K‐ treatment, where potassium deficiency appeared to inhibit stomatal responsiveness, resulting in slower dynamics even under drought conditions (Figure 5e,g). No significant differences were detected for the maximum slope of conductance (*SL*_max_), for light versus shade (exp. 1), for the drought × potassium combinations (Exp. 2), or for opening and closing. (Figure 5b,d,f,h).

### 3.4 Relation Between Dynamic Parameters of Opening and Closing Stomata

**FIGURE 6:**
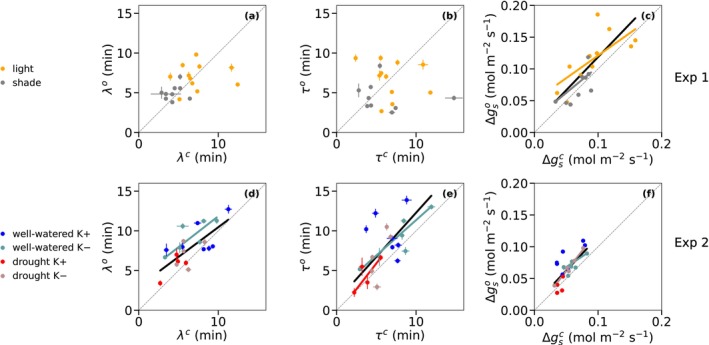
Relationships between stomatal conductance parameters measured during stomatal opening and closing. Experiment 1 (upper row): Relationships for light‐ and shade‐grown leaves for (a) Δ*g*_s_, (b) *t*_95_, and (c) *λ*. Experiment 2 (lower row): Relationships for well‐watered K+, well‐watered K–, drought K+, and drought K– treatments for (d) *λ*, (e) *τ*, and (f) Δ*g*_s_. The colours of the samples are the same as in the previous figures. The coloured lines represent the correlations for each treatment, and the black lines (solid) correspond to the correlations for all treatments combined for each experiment. The fitted lines (solid) use orthogonal distance regression to allow for uncertainties in *x* and *y*. Only significant lines are plotted. The dotted diagonal lines are 1:1 lines. Black lines are fits across all treatments within the experiments.

For Experiment 1, *λ* values during the closing phases of *g*_s_ were not correlated with *λ* during the opening phases of *g*_s_ (Figure 6a). Similarly, *τ* values were not correlated between opening and closing phases of *g*_s_ (Figure 6b), suggesting that distinct physiological mechanisms may control the lag time *λ* and response time *τ* of stomata during opening and closure phases of *g*_s_. The amplitude of conductance change (Δ*g*_s_) was positively correlated between opening and closing (Figure 6c). This suggests that only the amplitude correlates between opening and closing for Experiment 1. In Experiment 2, *λ*, *τ*, and Δ*g*_s_ showed clear linear relationships between opening and closing when all treatments were pooled (Figure 6d–f), indicating that changes in stomatal opening and closing increased proportionally, even though they are regulated independently. However, the relationships were less consistent for each individual treatment. A significant correlation between opening and closing was only observed for *λ* in well‐watered K– plants (Figure 6a). Correlations were observed for *τ* only in the well‐watered K– and drought K+ treatments, while other treatments showed no clear trend (Figure 6b). Correlations between opening and closing for Δ*g*_s_ were significant in all treatments except well‐watered K+ (Figure 6c). The lack of correlations likely reflects the small sample size within each treatment and the high variability among individual leaves. Nevertheless, when the data are considered collectively, the consistent linear trends observed across conditions in Experiment 2 suggest that *λ* and *τ* respond similarly to different environmental conditions for stomatal opening and closing processes, even though the underlying physiological mechanisms differ between opening and closing processes.

### 3.5 Gain and Loss of Carbon and Water due to Slow Stomatal Responses

**FIGURE 7:**
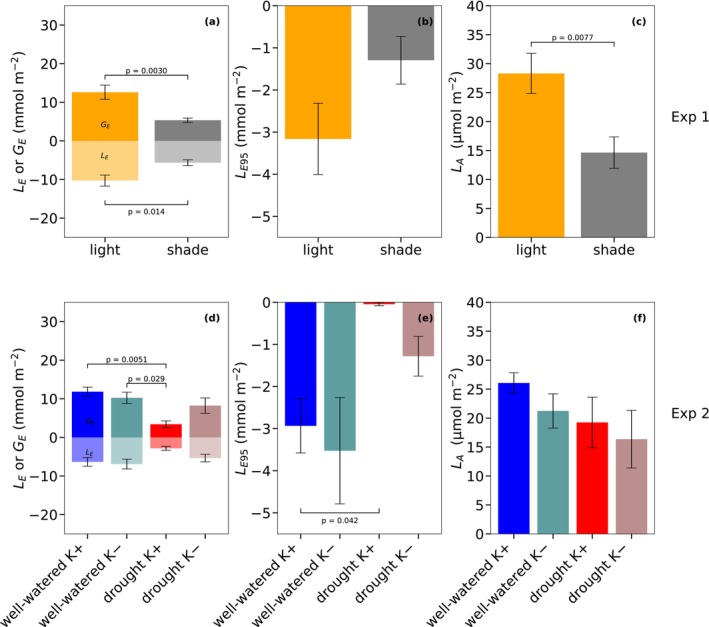
Water loss, water gain and carbon gain associated with stomatal dynamics following step changes in light for Experiment 1 (upper row) and Experiment 2 (lower row). (a, d) The amount of water lost due to delayed stomatal closure after a decrease in light (negative values, represented by light‐coloured bars, *L*_E_), and the amount of water conserved due to delayed stomatal opening after an increase in light (positive values, represented by dark‐coloured bars, *G*_E_). (b, e) The net assimilation lost due to delayed stomatal opening after a step increase in light (*L*_A_). (c, f). Excess water loss (*L*_E95_) caused by continued stomatal opening (overshoot) after CO_2_ assimilation (*A*_net_) reaches 95% of its final steady‐state value. Standard error (SE) is plotted in all panels. The scale in panel (c, f) differs from that in panel (a, d) to accommodate differences in the magnitude of water loss estimation.

Due to the progressive responses of stomata to light changes, the amount of water lost during stomatal closure (*L*_E_, Equation 6) and saved during opening (*G*_E_, Equation 8) differed between sunlit‐ and shade‐grown leaves (Figure 7a; Table S5). Sunlit‐grown leaves transpired significantly more water during closure (*L*_E_ = 10.29 ± 1.41 mmol m^−2^) than shade‐grown leaves (5.65 ± 0.75 mmol m^−2^), which was mainly due to larger Δ*g*_s_. Similarly, the delayed opening of stomata after an increase in light resulted in greater water conservation in sunlit‐grown leaves (*G*_E_ = 12.59 ± 1.84 mmol m^−2^) than in shade‐grown leaves (5.34 ± 0.56 mmol m^−2^). Stomatal conductance continued to increase even after assimilation had reached 95% of its final value, leading to additional water loss (*L*_E95_, Equation 9; Figure 7b). This excess water loss was not significantly different in sunlit‐grown leaves (3.16 ± 0.85 mmol m^−2^) than in shade‐grown leaves (1.29 ± 0.57 mmol m^−2^). Contrarily to *L*_E95_, assimilation limitation due to slow stomatal opening (*L*_A_, Equation 7; Figure 7c) was significantly lower in shade‐grown leaves (14.65 ± 2.70 μmol m^−2^) than in sunlit‐grown leaves (28.31 ± 3.47 μmol m^−2^; *p* = 0.0077; Table S5) (Figure 7c), consistent with the more responsive stomatal dynamics observed in shade‐grown leaves compared to light‐grown leaves (Figure 5). Conversely, no significant differences in net post‐illumination assimilation were observed between the two groups immediately after a reduction in light levels (data not shown). In Experiment 2, water status and potassium supply also influenced water loss and conservation during light transitions (Figure 7d,f; Table S5). Well‐watered K+ and K– plants showed slightly greater water loss during stomatal closure (*L*_E_) and significantly larger water savings during opening (*G*_E_) than droughted K+ plants (Figure 7d). Under drought, stomata showed smaller Δ*g*_s_ and shorter lag (*λ*) and response times (*τ*) during closing, which reduced water loss (*L*_E_). Stomata also showed shorter lag (*λ*) and response times (*τ*) during opening but the strong reduction in Δ*g*_s_ markedly increased limitation of assimilation (*L*_A_). Additional water loss after assimilation reached 95% of its maximum (*L*_E95_) also depended on water status (Figure 7e). Absolute *L*_E95_ was only significantly higher in well‐watered K+ plants than in drought K+ plants. The latter showed almost no additional losses. The limitation of assimilation due to slow stomatal opening (*L*_A_), however, did not differ significantly among the four water × potassium treatments (Figure 7f). For example, low final stomatal conductance (*g*_s,end_) of drought K+ plants (Table S3) prevented any improvement in carbon gain despite their faster stomatal response than well‐watered K+ plants.

## 4 Discussion

### 4.1 Physiological Adaptations Underlie Reduced Steady‐State Stomatal Conductance in Shade‐Grown Leaves

Shade‐grown leaves exhibited a lower change in stomatal conductance after reopening (Δ*g*_s_) compared to sunlit‐grown leaves (Figure 4c), primarily due to a lower maximal stomatal conductance (*g*_s_) after reopening (Table S4). This aligns with previous findings (Gross et al. 1996; Gerardin et al. 2018; Urban et al. 2007; Durand et al. 2022; Freitas et al. 2023) and reflects the adaptations of shade‐grown leaves to environments where photosynthesis is primarily limited by light availability rather than CO_2_. Similar acclimation patterns have been observed along canopy light gradients in mature beech forests, where shade leaves display lower stomatal conductance than sunlit‐exposed leaves in the upper canopy (Bögelein et al. 2012; Rajsnerová et al. 2015). In shaded environments, stomatal conductance is reduced due to lower demand for CO_2_ by assimilation. Shade conditions are typically associated with both reduced stomatal density, larger stomatal size (Poorter et al. 2025) and lower steady‐state stomatal conductance (Drake et al. 2013; Lawson and Blatt 2014; Caine et al. 2023). We found, however, no differences in stomatal density or size between treatments (Table S7), which contrasts with several previous studies reporting lower stomatal density in shade‐grown *Fagus sylvatica* leaves (Cano et al. 2013; Bögelein et al. 2012; Rajsnerová et al. 2015). Stomatal opening is regulated by biochemical and osmotic processes (Lawson and Vialet‐Chabrand 2019). Lower activation of guard‐cell ion transport or hormonal regulation in shaded leaves could explain the reduced stomatal opening when light decreases. Shade‐tolerant species such as *Fagus sylvatica* are known to display high plasticity of leaf traits along light gradients (Scartazza et al. 2016), supporting their capacity to adjust to fluctuating light environments. Despite the higher stomatal conductance and higher LMA observed in light‐grown leaves, photosynthetic parameters (*V*_cmax_, *J*_max_, Γ*) and *R*_d_ remained similar between shade‐ and light‐grown leaves, in contrast with previous findings in mature forests, where photosynthetic parameters changed with height and hence light environment (Montpied et al. 2009; Parelle et al. 2006; Cano et al. 2013; Walker et al. 2014). Shade‐grown plants had only about 11% irradiance than sunlit‐grown plants during at least 1 year before the experiment. This light reduction might not have been strong enough to induce the structural or biochemical adjustments typically observed in natural understory conditions. Nevertheless, light exposure still influenced stomatal behaviour, which appears more sensitive than photosynthetic capacities to relatively small differences in irradiance.

### 4.2 Adaptive Responses to Transient Light Availability

Shade‐grown leaves exhibited faster stomatal kinetics, characterized by lower lag times (*λ*) and shorter response times (*τ*) during opening, while *SL*_max_ remained unchanged. The stability of *SL*_max_ despite these changes implies that Δ*g*_s_ and *τ* are functionally linked, potentially through adjustments in ion transporter activity or membrane dynamics that simultaneously constrain stomatal range and accelerate responses. In contrast, the distinct changes in *λ*, which reflect the initial signal perception and transduction delays (Sussmilch et al. 2019), possibly involve light‐modulated pathways regulating early signalling events rather than the mechanical components governing the capacity of stomatal movement. The observed reduced stomatal response times *λ* and *τ* in shade‐grown leaves may significantly improve photosynthetic performance during transient light events. By aligning with faster Rubisco activation rates characteristic of shade‐acclimated leaves (Küppers and Schneider 1993; Durand et al. 2022), these responsive stomatal dynamics could optimize photosynthetic efficiency during long sunflecks or during clusters of sunflecks (Vialet‐Chabrand et al. 2013; McAusland et al. 2016). Shade‐adapted plants hence profit more from ephemeral light conditions in terms of CO_2_ assimilation while minimizing water loss through conductance regulation (Lawson and Blatt 2014). Reduced *λ* and *τ* exhibited by *Fagus sylvatica* under shade conditions contrast with responses observed in light‐adapted species. *Nicotiana tabacum* (tobacco), classified as a light‐demanding species adapted to high irradiance (Yang et al. 2017), and *Betula platyphylla* (Asian white birch), a pioneer species known for colonizing disturbed habitats (Sumida and Komiyama 1997), exhibit slower stomatal responses under shade (Gerardin et al. 2018; Zhao and Li 2022). This divergence may reflect a specific adaptation of the shade‐tolerant *F. sylvatica* to understory environments, where light availability is highly limiting. While sun species or sunlit‐acclimated leaves generally display faster photosynthetic induction (Küppers and Schneider 1993; Durand et al. 2022; Kang et al. 2024), shade‐grown leaves of *F. sylvatica* achieve full photosynthetic induction in a shorter overall time, despite a slower initial induction rate (Durand et al. 2022). This suggests that enhanced stomatal reactivity in *F. sylvatica* saplings facilitates a faster transition to full photosynthetic activity under low‐light. The observed shade‐induced plasticity of stomatal responsiveness in *F. sylvatica* is consistent with broader ecological patterns, whereby shade‐tolerant species tend to exhibit greater phenotypic plasticity, particularly in traits such as leaf biomass allocation under combined environmental stresses compared to pioneer species (Wang et al. 2021; Holmgren et al. 2012). Similarly, leaf mass per area (LMA) plasticity has also been reported to be higher in shade‐tolerant species (Kull and Niinemets 1993).

### 4.3 Adaptive Responses for Water Conservation and Gas Exchange Efficiency

Plants from drier climates (Vico et al. 2011) or those exposed to drought (Gerardin et al. 2018; Durand et al. 2019; Zhao and Li 2022) typically exhibit faster stomatal responses. Our study on *Fagus sylvatica* aligns with earlier studies showing that drought‐exposed and potassium‐supplemented plants had increased responsiveness of stomatal movement. Leaves thereby prioritized rapid signal detection (*λ*) during opening as well as steady‐state adjustments (*τ*) during opening and closing, which mirrored the strategy of shade‐grown leaves. The maximum speed of stomatal opening (*SL*_max_) remained largely unchanged under all treatments within each experiment. The increased responsiveness of *F. sylvatica* under moderate drought is consistent with observations in other species, including *Nicotiana tabacum* (Gerardin et al. 2018), *Populus* spp. (Durand et al. 2019), and *Betula platyphylla* (Zhao and Li 2022), allowing each to reach a steady‐state more quickly and to lose less water after changes in irradiance (Figure 7).

### 4.4 Potassium Supplementation Modifies the Responsiveness of Stomatal Conductance Under Drought

Potassium is a key cation in the regulation of stomatal functioning. High potassium concentrations in guard cells promote water influx and subsequent stomatal opening (Inoue et al. 2020), while very low soil potassium levels can reduce stomatal aperture (Thiel and Wolf 1997; Singh and Reddy 2017). For example, potassium deficiency significantly reduced stomatal conductance in both *Brassica napus* and *Carya cathayensis* saplings (Jin et al. 2011; Lu et al. 2016). Whereas in olive trees, moderately low potassium concentrations lead to persistently open stomata (Arquero et al. 2006). However, to date, no study has specifically addressed how potassium availability influences stomatal dynamics, particularly the temporal parameters of stomatal responses (i.e., *λ* and *τ*). In our study, potassium deficiency of the soil significantly reduced leaf potassium concentrations (cf. Materials and Methods), bringing them close to the upper threshold of nutrient deficiency symptoms previously reported for *Fagus sylvatica* (4.75 g kg^−1^) (Raluca‐Elena et al. 2022). Consistent with the observations in olive trees, potassium deficiency increased initial stomatal conductance (*g*_s,start_) in our study (Table S4). The limitation in potassium was associated with an attenuated stomatal response to drought stress (Figures 4h and 5g). Specifically, the typical drought‐induced reduction of lag time *λ* and response time *τ* was less pronounced in potassium‐deficient plants compared to plants with sufficient potassium nutrition, likely because potassium deficiency itself constrained stomatal functioning and masked the drought response. This pattern aligns with previous studies showing that potassium availability enhances plant resilience under drought stress in *Nicotiana rustica* (Bahrami‐Rad and Hajiboland 2017). Under well‐watered conditions, however, no significant differences were observed between K+ and K− treatments, suggesting that a potassium deficiency might mask the impact of soil drought on stomatal kinetics. In addition to its role in stomatal regulation, potassium deficiency is known to negatively affect leaf photosynthesis and reduce soluble glucose levels, as observed in apple trees ( *Malus domestica* ) (Ladikou et al. 2025). A similar decline in photosynthetic capacity was observed in potassium‐deficient plants in our study (Table S6). Reduced photosynthetic activity and soluble sugar accumulation in epidermal and guard cells caused by potassium deficiency may lower the osmotic potential of potassium‐deficient plants, thereby limiting water fluxes between cells. This would decrease leaf water potential and increase hydraulic resistance in potassium‐deficient leaves (Graham and Ulrich 1972). Such osmotic and hydraulic limitations could slow guard cell volume changes and could ultimately increase the lag time (*λ*) and response time (*τ*) of stomatal movements. Our findings suggest that soil potassium deficiency during drought directly alters leaf mineral composition, thereby affecting the signalling and regulatory pathways that govern stomatal kinetics. Hence, in our case, plants under potassium deficiency showed responses similar to those of well‐watered plants. It might be interesting to study the mechanisms behind nutrient‐related changes to stomatal dynamics in the future.

### 4.5 Ratio Between Opening and Closing Kinetics and Their Implications for Carbon‐Water Fluxes Regulation

The mechanisms underlying differences in stomatal kinetics have often been related to differences in stomatal size in the literature (Drake et al. 2013; Kardiman and Ræbild 2018) and particularly at the inter‐specific level (Xiong et al. 2022; Wall et al. 2023), suggesting that smaller stomata would be faster and more responsive (Raven 2014). Our findings at the within‐species level show that neither stomatal size nor density correlated with dynamic parameters, which suggests that physiological adaptations rather than anatomical differences drive the observed variations in stomatal conductance responsiveness. The reduced lag time (*λ*) observed during stomatal opening and closing in shade‐grown and drought‐stressed leaves likely reflects enhanced signal transduction efficiency (Matthews et al. 2019). Shade‐grown leaves show lower response times (*τ*) only during opening. This highlights the independent regulation of lag time *λ* and response time *τ*. However, across all treatments, stomatal opening was consistently faster than stomatal closure, resulting in a stable opening‐to‐closing ratio of approximately 1.2–1.3 for all dynamic parameters (Δ*g*_s_, *SL*_max_, *λ*, *τ*). This conserved proportionality suggests that the hydromechanical and ionic mechanisms controlling aperture changes in *Fagus sylvatica* (inward potassium fluxes, anion channel activation, membrane depolarisation–repolarisation cycles, and the osmotic flow of water) operate in a tightly coordinated manner across structural and environmental contexts (Schroeder et al. 2001; Outlaw Jr 2003; Lawson and Blatt 2014; Saito and Uozumi 2019). A conserved proportionality is not always the case, for example, in *Nicotiana tabacum* , where both drought and shade significantly change the opening‐to‐closing ratios of the dynamic parameters (Gerardin et al. 2018). Faster opening reflects more rapid activation of ion uptake and turgor increase in guard cells, whereas closure is inherently slower due to delayed deactivation of proton pumps, different types of ion channels between opening and closing of stomata, as well as slower osmotic water release (Lawson and Vialet‐Chabrand 2019). The observed stable ratio preserved in both shade‐grown and sunlit‐grown leaves indicates that stomatal dynamics scale proportionally with steady‐state conductance and do not undergo pronounced asymmetry, such as observed in other species (Drake et al. 2013; Raven 2014; Kardiman and Ræbild 2018). Only the well‐watered K+ plants deviated from the otherwise stable ratio of 1.2–1.3: in this treatment, both opening and closure were slower, but Δ*g*_s_ increased markedly so that the opening‐to‐closing ratio rose to 1.6 ± 0.2 (Table S1). This high ratio reflects globally reduced stomatal responsiveness combined with an excessive reopening amplitude under favourable conditions. Stomata tended to open far more than required to reach maximum photosynthesis in well‐watered K+ plants, leading to disproportionately high water losses during the closure phase (*L*_E95_), an effect consistent with the strong water losses observed in non‐limiting conditions by McAusland et al. (2016). Such behaviour suggests that, when water and nutrients are abundant, *Fagus sylvatica* prioritises carbon capture over water conservation, even at the cost of substantial transient transpiration. This dynamic proportionality has functional consequences for water balance under fluctuating irradiance. A ratio close to 1 implies that the water saved during rapid opening after an increase in light (*G*_E_) nearly compensates the water lost during the slower closure phase after a light decrease (*L*_E_). Shade‐grown leaves, with lower *g*_s_ and smaller Δ*g*_s_, lose little water during closure but also save little during opening, consistent with their cooler, humid microclimate and lower evaporative demand (Bögelein et al. 2012; Lemoine et al. 2002). Sunlit‐grown leaves show larger *L*_E_ but also larger *G*_E_ simply because their higher *g*_s_ amplify the water costs, so that slow stomatal movement leads to less water loss during opening hence larger *G*_E_. Under drought, both *G*_E_ and *L*_E_ become very small, reflecting strong stomatal restriction and minimal evaporative fluxes. However, this comes at the price of sustained stomatal limitation on carbon uptake (high *L*_A_), revealing a clear prioritisation of water conservation over photosynthetic performance (Aranda et al. 2012; Cano et al. 2013; Pflug et al. 2018). Under realistic canopy fluctuations, stomatal dynamics directly affect carbon gain. During cloudflecks and long sunflecks, the rapid opening of stomata reduces transient stomatal limitation and enhances CO_2_ uptake at the onset of photosynthetic induction (Küppers and Schneider 1993; Urban et al. 2007; Durand et al. 2022). Shade‐grown leaves have the lowest *L*_A_, so they particularly benefit from the combination of fast photosynthetic induction and fast stomatal dynamics, enabling them to exploit short light opportunities that can represent 30%–60%, and sometimes up to 80%–90% of daily carbon gain in understory environments (Weber et al. 1985; Pfitsch and Pearcy 1989; Chazdon and Pearcy 1991; Tang et al. 2003). Sunflecks can hence contribute significantly to annual photosynthesis in understory plants, ranging from 9% to 46% in low to high PPFD sites (Way and Pearcy 2012). Sunlit‐grown leaves, in contrast, experience larger water losses during closure but maintain overall neutral water loss and water conservation because *G*_E_ compensates *L*_E_ when integrated across a complete opening and closing cycle. Droughted plants exhibit the opposite strategy: rapid stomatal closure limits *L*_E_ but also leads to strong CO_2_ limitation (*L*_A_) for photosynthesis, with no further water loss after reaching 95% of assimilation (*L*_EA95_), resulting in reduced assimilation throughout the entire stomatal opening phase. It is interesting to note that photosynthesis can remain functional for seconds up to minutes after a sunfleck (post‐illumination phase), a phenomenon previously documented in *Fagus sylvatica* (Küppers and Schneider 1993). The authors also found stronger post‐illumination effects in shade‐grown leaves compared to sunlit‐grown leaves. This post‐illumination CO_2_ uptake can then partly compensate for slow stomatal closure. We did not observe such post‐illumination photosynthesis in our leaves, where the latter rapidly declined after reduction of irradiance. The dynamic responses of stomatal conductance and their underlying biochemical and osmotic mechanisms rather than anatomical differences explain how beech saplings maintain functional carbon‐water equilibrium across heterogeneous and fluctuating environments. The descriptions provide a mechanistic foundation for understanding performance of beech under future scenarios of increased light variability and water scarcity in the field. The dynamics of stomatal conductance have direct ecological implications. Under fluctuating irradiance dominated by sunflecks or cloudflecks, shade leaves maximise carbon gain when light is available but sacrifice water‐use efficiency (iWUE) during stomatal closure. Droughted leaves sustain high iWUE but lose opportunities for higher photosynthetic activity. Such contrasting strategies influence carbon–water trade‐offs at the seedling stage, a critical bottleneck for beech regeneration under increasingly warm and water‐limited conditions (Robson et al. 2009; Hommel et al. 2014; Martinez del Castillo et al. 2022). Our findings suggest that future increases in drought frequency may shift the balance in young *Fagus sylvatica* from a light‐driven, opportunistic strategy towards a more conservative hydraulic behaviour, with consequences for the success of establishment in shaded forest understories.

## Author Contributions

Yasin Gundesli, Oliver Brendel, Emilie Joetzjer, and Matthias Cuntz designed the experiment. Yasin Gundesli, Didier Le Thiec, David Combemale, Oliver Brendel, and Cyril Buré conducted the experiment. Yasin Gundesli, Oliver Brendel, Emilie Joetzjer, and Matthias Cuntz analysed the data and interpreted the results. Yasin Gundesli wrote the manuscript together with Oliver Brendel, Emilie Joetzjer, and Matthias Cuntz. Didier Le Thiec revised the manuscript. All authors approved the final version.

## Funding

Funding was provided by the French National Research Agency (ANR) ANR‐21‐CE02‐0033‐01, and grants (SlowStom, DONUTS and METHAFOR) overseen by ANR as part of the “Investissements d'Avenir” program (ANR‐11‐LABX‐0002‐01, Lab of Excellence ARBRE) and by the interdisciplinary program ARTEMIS of Lorraine Université d'Excellence (ANR‐15‐IDEX‐04‐LUE). Y. Gundesli profited from a PhD grant of the Université de Lorraine.

## Supporting information


**Figure S1:** Relationships between stomatal dynamic parameters and water as well as CO_2_ losses and gains during opening and closing of stomata.
**Figure S2:** Meteorological conditions inside the greenhouse during the measurement period.
**Figure S3:** Drought application: intensity and duration in beech seedling experiments.
**Table S1:** Mean ratios ± standard errors of the parameters for stomatal closing over the parameters for stomatal opening.
**Table S2:** Mean ± standard errors of the parameters for stomatal opening.
**Table S3:** Mean ± standard errors of the parameters for stomatal closing.
**Table S4:** Mean ± standard errors of the steady‐state stomatal conductance.
**Table S5:** Mean ± standard errors of the loss of water (*L*_E_) during closing, gain of water (*G*_E_) and water lost after assimilation reached 95% of its final value (*L*_E95_) during opening and limitation of assimilation by slow stomatal conductance response during opening (*L*_A_).
**Table S6:** Mean ratios ± standard errors of photosynthetic parameters. Significant differences between treatments are indicated by different letters within each treatment group.
**Table S7:** Mean ratios ± standard errors of anatomical parameters of stomata. LMA, leaf mass area; SD, stomatal density; SL, stomatal length; SW, stomatal width.

## Data Availability

The data that supports the findings of this study are available in the Supporting Information of this article.
