# Supplementary material for: Shade and Drought Shape Stomatal Kinetics in Beech Saplings
Source: Physiol Plant. 2026 Apr 3;178(2):e70856. doi: 10.1111/ppl.70856 (PMC13049098; doi:10.1111/ppl.70856)
Supplement: Supplementary file 1 — Figure S1: Relationships between stomatal dynamic parameters and water as well as CO2 losses and gains during opening and closing of stomata. Figure S2: Meteorological conditions inside the greenhouse during the measurement period. Figure S3: Drought application: intensity and duration in beech seedling experiments. Table S1: Mean ratios ± standard errors of the parameters for stomatal closing over the parameters for stomatal opening. Table S2: Mean ± standard errors of the parameters for stomatal opening. Table S3: Mean ± standard errors of the parameters for stomatal closing. Table S4: Mean ± standard errors of the steady‐state stomatal conductance. Table S5: Mean ± standard errors of the loss of water (L E) during closing, gain of water (G E) and water lost after assimilation reached 95% of its final value (L E95) during opening and limitation of assimilation by slow stomatal conductance response during opening (L A). Table S6: Mean ratios ± standard errors of photosynthetic parameters. Significant differences between treatments are indicated by different letters within each treatment group. Table S7: Mean ratios ± standard errors of anatomical parameters of stomata. LMA, leaf mass area; SD, stomatal density; SL, stomatal length; SW, stomatal width. [file PPL-178-e70856-s001.docx]

**Shade and Drought Shape Stomatal Kinetics in Beech Saplings**

**Yasin Gundesli, Emilie Joetzjer, Oliver Brendel, Didier Le Thiec, David Combemale, Cyril Buré, Matthias Cuntz**

Université de Lorraine, AgroParisTech, INRAE, UMR Silva, Nancy, France

Correspondance : Matthias Cuntz ([matthias.cuntz@inrae.fr](mailto:matthias.cuntz@inrae.fr))

Keywords:

*drought stress, European beech, light acclimation, potassium deficiency, stomatal dynamics*

**Supporting information**


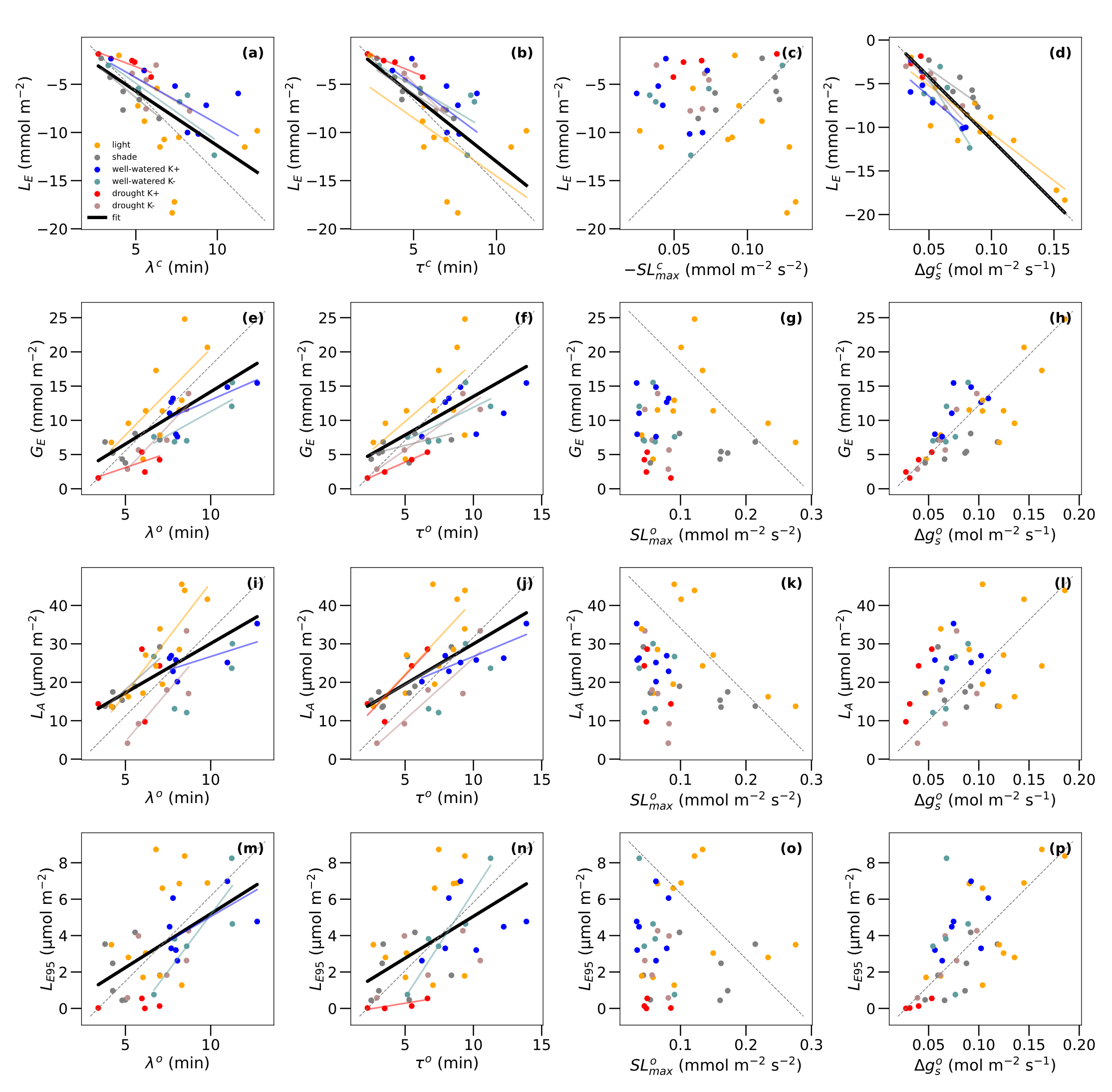


**Figure S1** Relationships between stomatal dynamic parameters and water as well as CO2 losses and gains during opening and closing of stomata. The first row shows the relationship between the loss of water during stomatal closure (*L_E_*) and (a) lag time (*λ*), (b) response time (*τ*), (c) maximal slope (*SLₘₐₓ*), and (d) the amplitude of stomatal conductance (Δ*g_s_*). The second row shows the relationship between the gain of water during stomatal opening (*G_E_*) and (e) lag time (*λ*), (f) response time (*τ*), (g) maximal slope (*SLₘₐₓ*), and (h) the amplitude of stomatal conductance (Δ*g_s_*). The third row shows the relationship between the limitation of assimilation during stomatal opening (*L_A_*) and (i) lag time (*λ*), (j) response time (*τ*), (k) maximal slope (*SL_max_*), and (l) the amplitude of stomatal conductance (Δ*g_s_*). The last row shows the relationship between excess water loss due to stomatal overshoot after assimilation reaches 95% (*L_E95_*) and (m) lag time (*λ*), (n) response time (*τ*), (o) maximal slope (*SLₘₐₓ*), and (p) the amplitude of stomatal conductance (Δ*g_s_*). Sample sizes and colours are as in the previous figures. Solid lines represent linear regressions. The black solid line uses all data from all treatments while the coloured lines are for the individual treatments. Only significant lines are plotted. Dotted lines indicate the 1:1 lines.

**
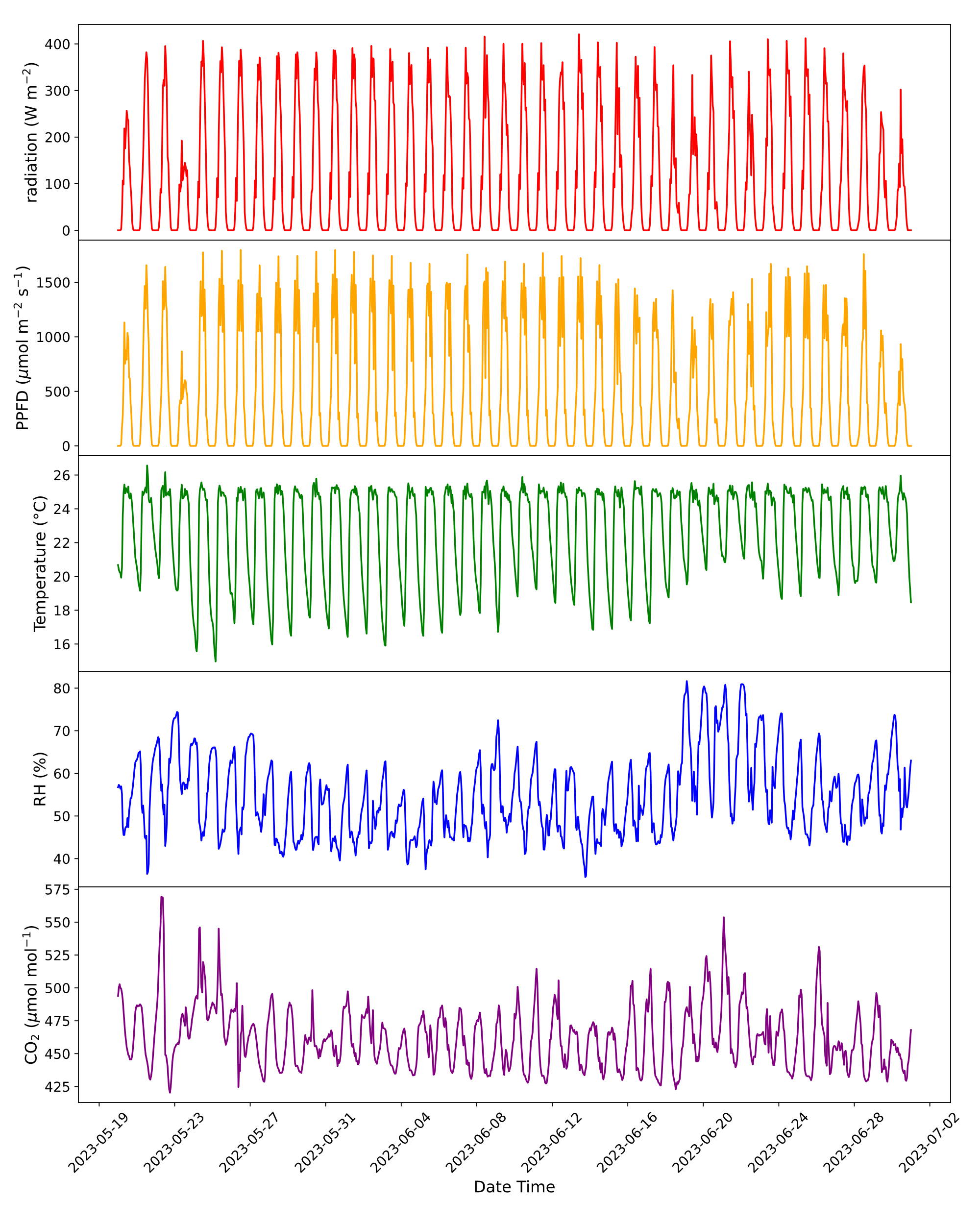
**

**Figure S2** Meteorological conditions inside the greenhouse during the measurement period. (a) Global radiation (W m⁻²), (b) Photosynthetic photon flux density (PPFD, µmol m⁻² s⁻¹), (c) Temperature (°C), (d) Relative humidity (%), and (e) Atmospheric CO₂ concentration (µmol mol⁻¹).

**
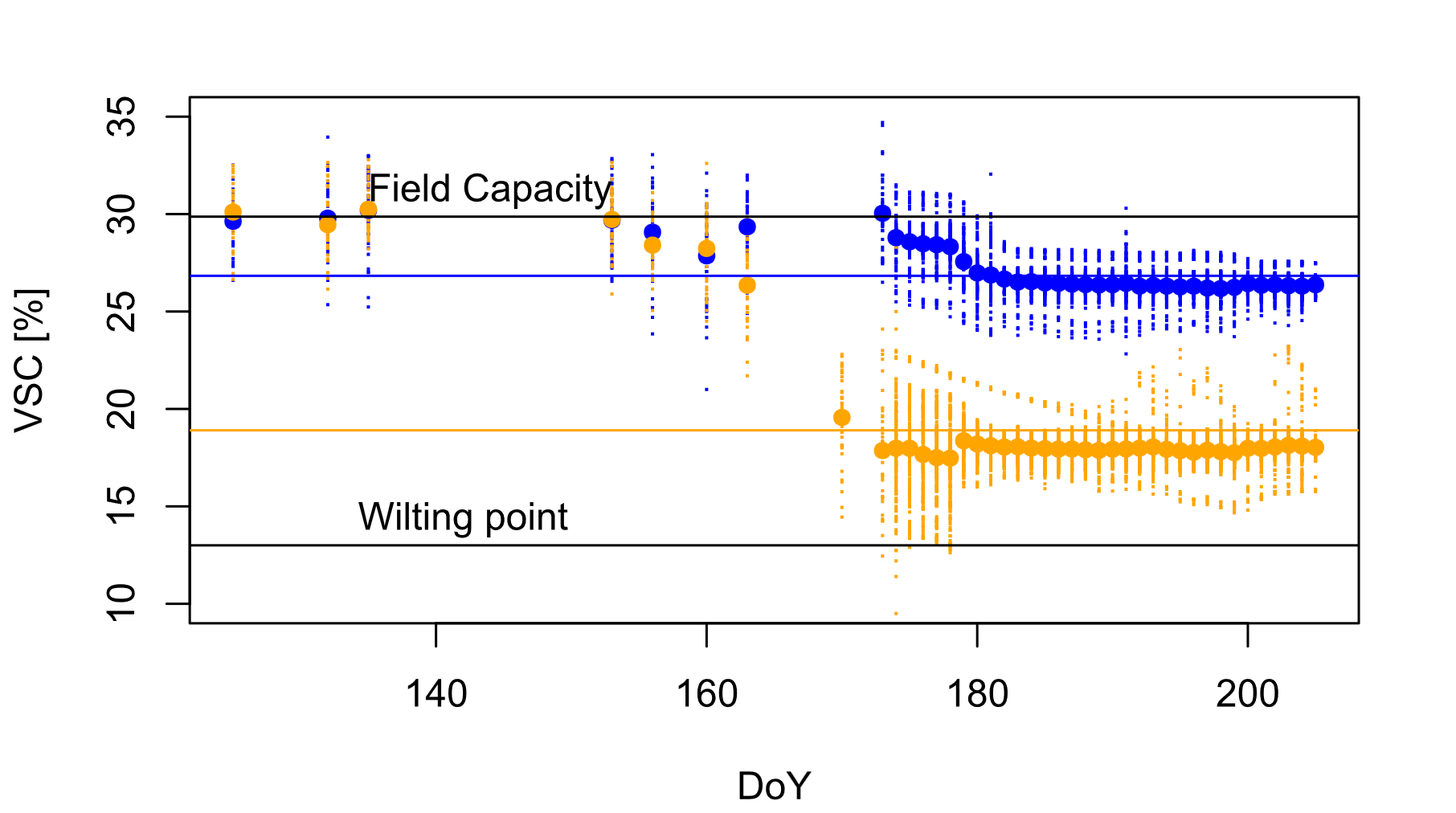
**

**Figure S3** Drought application: intensity and duration in beech sapling experiments. Volumetric Soil water Content (VSC, %) over time (DOY: Day of Year). Blue points represent well-watered plants, orange points represent drought-stressed plants. Small points indicate individual measurements; large points indicate the mean per group. The solid lines represent the target VSC values used to reach specific Relative Water Content (RWC, %) levels: 26.83% VSC for 82% RWC in well-watered plants, and 18.9% VSC for 35% RWC for drought-stressed plants. The upper black line indicates field capacity; the lower black line indicates the wilting point.

**Table S1** Mean ratios ± standard errors of the parameters for stomatal closing over the parameters for stomatal opening. Significant differences between treatments are indicated by different letters within each treatment group. Comparisons between light and shade were performed using a t-test (p < 0.05). For the well-watered K+, well-watered K−, drought K+, and drought K− treatments, a two-way ANOVA was performed, and post hoc comparisons were conducted using Tukey’s HSD test (p < 0.05), statistical significance of ANOVA is indicated as *p < 0.05, **p < 0.01, ***p < 0.001; ns denotes non-significant differences. Pairwise tests between stomatal opening and closing parameters were conducted to assess asymmetry. Statistical significance of pairwise test is indicated just after the mean ratios ± standard errors as *p < 0.05, **p < 0.01, ***p < 0.001; the absence of * just after the mean ratios ± standard errors indicate that it is not significant.

| **Treatment** | ${\boldsymbol{\lambda}^{\boldsymbol{o}}}/{\boldsymbol{\lambda}^{\boldsymbol{c}}}$ | ${\boldsymbol{\tau}^{\boldsymbol{o}}}/{\boldsymbol{\tau}^{\boldsymbol{c}}}$ | ${\boldsymbol{SL}_{\boldsymbol{max}}^{\boldsymbol{o}}}/{\boldsymbol{SL}_{\boldsymbol{max}}^{\boldsymbol{c}}}$ | ${\boldsymbol{\Delta g}_{\boldsymbol{s}}^{\boldsymbol{o}}}/{\boldsymbol{\Delta g}_{\boldsymbol{s}}^{\boldsymbol{c}}}$ | ${\boldsymbol{t}_{\boldsymbol{95}}^{\boldsymbol{o}}}/{\boldsymbol{t}_{\boldsymbol{95}}^{\boldsymbol{c}}}$ |
| --- | --- | --- | --- | --- | --- |
| light | 1.1 ± 0.1a | 1.2 ± 0.3a | 1.5 ± 0.2a | 1.3 ± 0.1a* | 1.2 ± 0.2a |
| shade | 1.2 ± 0.1a | 0.95 ± 0.19a | 1.5 ± 0.3a | 1.1 ± 0.09a | 0.98 ± 0.17a |
| well-watered K+ | 1.3 ± 0.2a | 1.6 ± 0.3a* | 1.2 ± 0.2a | 1.6 ± 0.2a** | 1.5 ± 0.2a |
| well-watered K− | 1.5 ± 0.2a** | 1.2 ± 0.1a | 0.98 ± 0.072a | 1.2 ± 0.07ab* | 1.3 ± 0.1a* |
| drought K+ | 1.2 ± 0.1a | 1.2 ± 0.2a | 0.8 ± 0.08a | 0.95 ± 0.12b | 1.2 ± 0.1a |
| drought K− | 1.2 ± 0.1a | 1.2 ± 0.2a | 1.2 ± 0.3a | 1.2 ± 0.03ab** | 1.2 ± 0.2a |
| Water stress effect | ns | ns | ns | ns | ns |
| Potassium stress effect | ns | ns | ns | ns | ns |
| Water:potassium stress effect | ns | ns | ns | * | ns |
| Overall | 1.2 ± 0.06* | 1.2 ± 0.1 | 1.3 ± 0.09* | 1.3 ± 0.06*** | 1.2 ± 0.09 |

**Table S2** Mean ± standard errors of the parameters for stomatal opening. Significant differences be-tween treatments are indicated by different letters within each treatment group. Comparisons between light and shade were performed using a t-test (p < 0.05). For the well-watered K+, well-watered K−, drought K+, and drought K− treatments, a two-way ANOVA was per-formed, and post hoc comparisons were conducted using Tukey’s HSD test (p < 0.05), statistical significance of ANOVA is indicated as *p < 0.05, **p < 0.01, ***p < 0.001; ns denotes non-significant differences.

| **Treatment** | $\boldsymbol{\lambda}^{\boldsymbol{o}}$  **(min)** | $\boldsymbol{\tau}^{\boldsymbol{o}}$  **(min)** | $\boldsymbol{SL}_{\boldsymbol{max}}^{\boldsymbol{o}}$  **(mmol m^−2^ s^−2^)** | $\boldsymbol{\Delta g}_{\boldsymbol{s}}^{\boldsymbol{o}}$  **(mol m^−2^ s^−1^)** | $\boldsymbol{t}_{\boldsymbol{95}}^{\boldsymbol{o}}$  **(min)** |
| --- | --- | --- | --- | --- | --- |
| light | 7 ± 0.5a | 6.7 ± 0.7a | 0.12 ± 0.022a | 0.12 ± 0.012a | 27 ± 2.5a |
| shade | 5 ± 0.3b | 4.5 ± 0.6b | 0.12 ± 0.021a | 0.072 ± 0.0085b | 18 ± 2.1b |
| well-watered K+ | 9 ± 0.8a | 9.7 ± 1a | 0.056 ± 0.0080a | 0.082 ± 0.0076a | 38 ± 3.4a |
| well-watered K− | 9.4 ± 0.8a | 8.8 ± 1.2ab | 0.054 ± 0.0087a | 0.071 ± 0.0047a | 36 ± 4.3ab |
| drought K+ | 5.6 ± 0.8b | 4.5 ± 0.9b | 0.057 ± 0.0096a | 0.038 ± 0.0057b | 19 ± 3.5b |
| drought K− | 7.1 ± 0.7ab | 6.9 ± 1.4ab | 0.066 ± 0.0071a | 0.069 ± 0.0097ab | 27 ± 4.8ab |
| Water stress effect | * | * | ns | * | * |
| Potassium stress effect | ns | ns | ns | ns | ns |
| Water:potassium stress effect | ns | ns | ns | * | ns |

**Table S3** Mean ± standard errors of the parameters for stomatal closing. Significant differences between treatments are indicated by different letters within each treatment group. Comparisons between light and shade were performed using a t-test (p < 0.05). For the well-watered K+, well-watered K−, drought K+, and drought K− treatments, a two-way ANOVA was performed, and post hoc comparisons were conducted using Tukey’s HSD test (p < 0.05), statistical significance of ANOVA is indicated as *p < 0.05, **p < 0.01, ***p < 0.001; ns denotes non-significant differences.

| **Treatment** | $\boldsymbol{\lambda}^{\boldsymbol{c}}$  **(min)** | $\boldsymbol{\tau}^{\boldsymbol{c}}$  **(min)** | $\left\vert\boldsymbol{SL}_{\boldsymbol{max}}^{\boldsymbol{c}} \right\vert$  **(mmol m^−2^ s^−2^)** | $\boldsymbol{\Delta g}_{\boldsymbol{s}}^{\boldsymbol{c}}$  **(mol m^−2^ s^−1^)** | $\boldsymbol{t}_{\boldsymbol{95}}^{\boldsymbol{c}}$  **(min)** |
| --- | --- | --- | --- | --- | --- |
| light | 7.3 ± 0.8a | 6.8 ± 0.8a | 0.090 ± 0.011a | 0.093 ± 0.012a | 28 ± 3.1a |
| shade | 4.4 ± 0.4b | 6 ± 1.2a | 0.085 ± 0.012a | 0.069 ± 0.0063a | 22 ± 3.6a |
| well-watered K+ | 7.7 ± 1a | 6.7 ± 0.7ab | 0.050 ± 0.0066a | 0.053 ± 0.0069a | 28 ± 2.9a |
| well-watered K− | 6.6 ± 1a | 7.6 ± 1.3a | 0.060 ± 0.015a | 0.060 ± 0.0051a | 29 ± 4.3a |
| drought K+ | 4.6 ± 0.7a | 3.7 ± 0.7b | 0.074 ± 0.016a | 0.040 ± 0.0024a | 16 ± 2.7a |
| drought K− | 6.1 ± 0.6a | 5.4 ± 0.5ab | 0.063 ± 0.0065a | 0.055 ± 0.0074a | 22 ± 1.8a |
| Water stress effect | ns | * | ns | ns | * |
| Potassium stress effect | ns | ns | ns | ns | ns |
| Water:potassium stress effect | ns | ns | ns | ns | ns |

**Table S4** Mean ± standard errors of the steady-state stomatal conductance. Significant differences between treatments are indicated by different letters within each treatment group. Comparisons between light and shade were performed using a t-test (p < 0.05). For the well-watered K+, well-watered K−, drought K+, and drought K− treatments, a two-way ANOVA was performed, and post hoc comparisons were conducted using Tukey’s HSD test (p < 0.05), statistical significance of ANOVA is indicated as *p < 0.05, **p < 0.01, ***p < 0.001; ns denotes non-significant differences.

| **Treatment** | $\boldsymbol{g}_{\boldsymbol{s,start}}^{\boldsymbol{o}}$  **(mol m^−2^ s^−1^)** | $\boldsymbol{g}_{\boldsymbol{s,end}}^{\boldsymbol{o}}$  **(mol m^−2^ s^−1^)** | $\boldsymbol{g}_{\boldsymbol{s,start}}^{\boldsymbol{c}}$  **(mol m^−2^ s^−1^)** | $\boldsymbol{g}_{\boldsymbol{s,end}}^{\boldsymbol{c}}$  **(mol m^−2^ s^−1^)** |
| --- | --- | --- | --- | --- |
| light | 0.071 ± 0.0058a | 0.19 ± 0.014a | 0.16 ± 0.016a | 0.069 ± 0.0055a |
| shade | 0.066 ± 0.0059a | 0.14 ± 0.012b | 0.13 ± 0.011a | 0.066 ± 0.0052a |
| well-watered K+ | 0.059 ± 0.011ab | 0.14 ± 0.017a | 0.12 ± 0.015ab | 0.064 ± 0.01ab |
| well-watered K− | 0.074 ± 0.0049a | 0.14 ± 0.0052a | 0.14 ± 0.007a | 0.076 ± 0.0052a |
| drought K+ | 0.029 ± 0.0048b | 0.067 ± 0.0096b | 0.071 ± 0.0052b | 0.031 ± 0.0048b |
| drought K− | 0.05 ± 0.007ab | 0.12 ± 0.016ab | 0.11 ± 0.014ab | 0.052 ± 0.007ab |
| Water stress effect | * | ** | * | ** |
| Potassium stress effect | * | ns | * | ns |
| Water:potassium stress effect | ns | ns | ns | ns |

**Table S5** Mean ± standard errors of the loss of water (*L_E_*) during closing, gain of water (*G_E_*) and water lost after assimilation reached 95% of its final value (*L_E95_*) during opening and limitation of assimilation by slow stomatal conductance response during opening (*L_A_*). Significant differences between treatments are indicated by different letters within each treatment group. Comparisons between light and shade were performed using a t-test (p < 0.05). For the well-watered K+, well-watered K−, drought K+, and drought K− treatments, a two-way ANOVA was performed, and post hoc comparisons were conducted using Tukey’s HSD test (p < 0.05), statistical significance of ANOVA is indicated as *p < 0.05, **p < 0.01, ***p < 0.001; ns denotes non-significant differences.

| **Treatment** | ***L_E_* (mmol m^−2^)** | ***G_E_* (mmol m^−2^)** | ***L_A_* (mmol m^−2^)** | ***L_E95_* (mmol m^−2^)** |
| --- | --- | --- | --- | --- |
| light | −10.28 ± 1.41a | 12.59 ± 1.84a | 28.31 ± 3.47a | -3.16 ± 0.85a |
| shade | −5.65 ± 0.75b | 5.34 ± 0.56b | 14.65 ± 2.7b | -1.29 ± 0.57a |
| well-watered K+ | −6.34 ± 1.13a | 11.83 ± 1.18a | 26.06 ± 1.77a | -2.93 ± 0.64a |
| well-watered K− | −6.91 ± 1.26a | 10.24 ± 1.47a | 21.23 ± 2.96a | -3.52 ± 1.26a |
| drought K+ | −2.83 ± 0.51a | 3.4 ± 0.85b | 19.25 ± 4.36a | -0.04 ± 0.04b |
| drought K− | −5.35 ± 0.97a | 8.23 ± 2ab | 16.36 ± 4.97a | -1.28 ± 0.47a |
| Water stress effect | ns | * | ns | ** |
| Potassium stress effect | ns | ns | ns | ns |
| Water:potassium stress effect | ns | ns | ns | * |

**Table S6** Mean ratios ± standard errors of photosynthetic parameters. Significant differences between treatments are indicated by different letters within each treatment group. Comparisons between light and shade were performed using a t-test (p < 0.05). For the well-watered K+, well-watered K−, drought K+, and drought K− treatments, a two-way ANOVA was performed, and post hoc comparisons were conducted using Tukey’s HSD test (p < 0.05), statistical significance of ANOVA is indicated as *p < 0.05, **p < 0.01, ***p < 0.001; ns denotes non-significant differences.

| **Treatment** | ***V_cmax_***  **(µmol m^−2^ s^−1^)** | ***J_max_***  **(µmol m^−2^ s^−1^)** | ***A_max_* (µmol m^−2^ s^−1^)** | ***A_sat_***  **(µmol m^−2^ s^−1^)** | **Γ^*^**  **(µmol m^−2^ s^−1^)** | **R_d_**  **(µmol m^−2^ s^−1^)** |
| --- | --- | --- | --- | --- | --- | --- |
| light | 51 ± 2a | 90 ± 3a | 18 ± 1a | 16 ± 1a | 51 ± 1a | −0.64 ± 0.091a |
| shade | 47 ± 2a | 86 ± 4a | 18 ± 1a | 15 ± 1a | 49 ± 1a | −0.56 ± 0.041a |
| well-watered K+ | 36 ± 1.3a | 73 ± 2.3a | 16 ± 0.53a | 11 ± 0.47a | 48 ± 1.3a | −0.71 ± 0.26a |
| well-watered K− | 29 ± 0.82a | 56 ± 4.5a | 12 ± 1.1a | 8.9 ± 0.42a | 51 ± 0.91a | −0.85 ± 0.095a |
| drought K+ | 35 ± 3.4a | 66 ± 7a | 14 ± 1.5a | 10 ± 1a | 51 ± 2.1a | −0.84 ± 0.11a |
| drought K− | 29 ± 2.8a | 55 ± 6.2a | 12 ± 1.3a | 8.8 ± 1a | 54 ± 0.25a | −0.28 ± 0.17a |
| Water stress effect | ns | ns | ns | ns | ns | ns |
| Potassium stress effect | * | * | * | * | ns | ns |
| Water:potassium stress effect | ns | ns | ns | ns | ns | * |

**Table S7** Mean ratios ± standard errors of anatomical parameters of stomata. SD: stomatal density, SL: Stomatal length, SW: stomatal width, LMA: Leaf mass area. Significant differences between treatments are indicated by different letters within each treatment group. Comparisons between light and shade were performed using a t-test (p < 0.05). For the well-watered K+, well-watered K−, drought K+, and drought K− treatments, a two-way ANOVA was performed, and post hoc comparisons were conducted using Tukey’s HSD test (p < 0.05), statistical significance of ANOVA is indicated as *p < 0.05, **p < 0.01, ***p < 0.001; ns denotes non-significant differences.

| **Treatment** | **SD**  **(stomata mm^−2)^** | **SL**  **(µm)** | **SW**  **(µm)** | **LMA (g m^−2^)** |
| --- | --- | --- | --- | --- |
| light | 275.52 ± 27.27a | 22.20 ± 1.02a | 16.21 ± 0.71a | 61.32 ± 2.18a |
| shade | 278.12 ± 23.87a | 21.02 ± 0.54a | 15.81 ± 0.41a | 46.47 ± 2.30b |
| well-watered K+ | 316.61 ± 22.84a | 19.90 ± 0.49a | 16.08 ± 0.81a | 59.57 ± 2.39a |
| well-watered K− | 291.58 ± 18.45a | 19.47 ± 0.66a | 15.92 ± 0.54a | 56.01 ± 2.96a |
| drought K+ | 286.66 ± 20.93a | 18.47 ± 0.68a | 15.70 ± 0.46a | 55.85 ± 2.74a |
| drought K− | 313.97 ± 17.84a | 19.19 ± 1.16a | 16.26 ± 0.59a | 55.81 ± 3.90a |
| Water stress effect | ns | ns | ns | ns |
| Potassium stress effect | ns | ns | ns | ns |
| Water:potassium stress effect | ns | ns | ns | ns |
